# The routine use of LCD-Array hybridisation technique for HPV subtyping in the diagnosis of penile carcinoma compared to other methods

**DOI:** 10.1186/s12894-022-00962-4

**Published:** 2022-01-29

**Authors:** Ria Winkelmann, Katrin Bankov, Jens von der Grün, Jindrich Cinatl, Peter J. Wild, Stefan Vallo, Melanie Demes

**Affiliations:** 1 Dr. Senckenberg Institute of Pathology, University of Frankfurt, Theodor-Stern-Kai 7, 60596 Frankfurt am Main, Germany; 2 Department of Radiation Oncology, University of Frankfurt, Theodor-Stern-Kai 7, 60596 Frankfurt am Main, Germany; 3 Institute of Medical Virology, University of Frankfurt, Theodor-Stern-Kai 7, 60596 Frankfurt am Main, Germany

**Keywords:** Urological oncology, Viral infection, Viral oncology, Screening, HPV infection, Penile carcinomas, Sanger Sequencing, p16, LCD-Array

## Abstract

**Background:**

Routine human papillomavirus (HPV) testing is performed in cervival cancer and is required for classification of some head and neck cancers. In penile cancer a statement on HPV association of the carcinoma is required. In most cases p16 immunohistochemistry as a surrogate marker is applied in this setting. Since differing clinical outcomes for HPV positive and HPV negative tumors are described we await HPV testing to be requested more frequently by clinicians, also in the context of HPV vaccination, where other HPV subtypes are expected to emerge.

**Method:**

Therefore, a cohort of archived, formalin-fixed paraffin embedded (FFPE) penile neoplasias was stained for p16 and thereafter tested for HPV infection status via PCR based methods. Additionally to Sanger sequencing, we chose LCD-Array technique (HPV 3.5 LCD-Array Kit, Chipron; LCD-Array) for the detection of HPV in our probes expecting a less time consuming and sensitive HPV test for our probes.

**Results:**

We found that LCD-Array is a sensitive and feasible method for HPV testing in routine diagnostics applicable to FFPE material in our cohort. Our cohort of penile carcinomas and carcinomas in situ was associated with HPV infection in 61% of cases. We detected no significant association between HPV infection status and histomorphological tumor characteristics as well as overall survival.

**Conclusions:**

We showed usability of molecular HPV testing on a cohort of archived penile carcinomas. To the best of our knowledge, this is the first study investigating LCD-Array technique on a cohort of penile neoplasias.

**Supplementary Information:**

The online version contains supplementary material available at 10.1186/s12894-022-00962-4.

## Background

Infection by human papillomavirus (HPV) is associated with a variety of cancers including anal squamous cell carcinomas (SCC), oropharyngeal cancers, cervical cancers, vulvar and vaginal cancers as well as penile carcinomas [1]. Penile neoplasia is a rare disease entity in Europe and the USA with an incidence of less than 1/100.000 [2]. The WHO classification of diseases describes HPV-related and non-HPV-related penile squamous cell carcinomas [3]. HPV infection rates in penile cancers vary from study to study from about one third to two thirds [4]. Penile carcinomas not related to HPV are thought to be associated with numerous factors, for example lack of neonatal circumcision and cigarette smoking [5]. The current S3 guideline for penile carcinomas states that it is necessary to give a statement on HPV association of the carcinoma [6]. The outcome of HPV-associated cancers, such as anal squamous cell carcinomas and oropharyngeal carcinomas, as well as penile carcinomas differ from non-HPV-related cancers [7, 8]. In routine diagnostics an antibody against p16 is recommended for the detection of HPV infections in penile carcinomas [9]. p16 serves as a surrogate marker to detect a possible infection by HPV. Stainings are mostly positive in case of infection by high risk HPV subtypes. Interpreting the staining result needs some experience to not over interpret weak or mosaic-like staining patterns as clear-cut positive results. In uncertain cases, another method that can be applied on formalin fixed paraffin embedded (FFPE) tissue specimen is needed. Additionally, due to differences in clinical outcomes for HPV positive and HPV negative tumors reliable HPV testing may become requested more frequently by clinicians, also in the context of HPV vaccination, where other HPV subtypes are expected to emerge.

We used p16 immunohistochemistry, Sanger sequencing and the LCD-Array hybridisation technique (Chipron) for HPV detection on a cohort of penile squamous cell carcinomas, carcinomas in situ and peritumoral tissue and compared the sensitivity and specificity, respectively. We chose LCD-Array hybridisation technique (Chipron) in direct comparison to Sanger sequencing expecting reduced hands-on time and easy interpretation of the results. To shed light on the question of which test is the most efficient one for daily routine, we compared the above mentioned three distinct tests. To our knowledge, this is the first study testing LCD array technique on a cohort of FFPE material of penile neoplasias.

## Methods

### Patient characteristics

Between 2002 and 2017 70 cases with sufficient FFPE material were identified with penile squamous cell carcinomas, squamous cell carcinoma in situ (Cais) of the penis, extramammary Paget disease, focally invasive as well as basal cell carcinoma of the penis. The paraffin blocks were provided by the Dr. Senckenberg Institute of Pathology, University Hospital, Frankfurt, Germany.

We systematically compared tumor and individual matching non-tumor probes for 69 of 70 cases. In a single case, a lymph node metastasis was investigated. Therefore, we applied a total of 139 tests, for one probe only metastatic material was available. In total, we investigated 62 invasive squamous cell carcinomas, 6 squamous cell carcinomas in situ, one basal cell carcinoma, and one extramammary Paget disease, focally invasive. Additionally, peritumoral epithelium was tested, wherever available. The peritumoral epithelium was macrodissected or taken from another block wherever available.

Median age at diagnosis was 65 years (range 41–85 years). The lesions were mostly located at the glans penis (n = 50), followed by the foreskin (n = 9) and shaft (n = 2). In 9 cases, the primary tumor localisation could not be determined due to either a large tumor size or incomplete documentation.

Penile carcinomas were staged and characterized according to TNM staging and the 8th edition of the classification system of the Union for International Cancer Control (UICC) [9]. The tumor classification is displayed in Table 1. Tumor size and infiltration depth were recorded for all tumors. In cases of incomplete excision, or undeterminable tumor size, the tumor measurements were not included. Infiltration depth was measured in mm from deepest infiltration to epithelium for all tumors.

**Table 1:** Characteristics of invasive squamous cell carcinomas of the penis (n = 60*)

Characteristics	n	%**
pTX	2	3
pT1a	23	38
pT1b	7	12
pT2	23	38
pT3	3	5
pT4	2	3
pNX	38	63
pN0	13	22
pN1, pN2, pN3	9	15
LX	1	2
L0	47	78
L1	12	20
VX	1	2
V0	54	90
V1	5	8
PnX	1	2
Pn0	53	88
Pn1	6	10
G1	12	20
G2	39	65
G3	9	15
Usual morphology	54	90
Basaloid morphology	6	10
Infiltrative pattern	38	63
Pushing borders	22	37

*In two cases only central biopsy probe or metastasis was present

**% may not add up to 100 because of rounding

Tissue samples and patient data used in this study were provided by the University Cancer Center Frankfurt (UCT). Written informed consent was obtained from all patients and the study was approved by the institutional review board of the UCT and the ethical committee at the University Hospital Frankfurt (project-number: SUG-02-2017) according to the declaration of Helsinki. For our studies, archived material was used in a double pseudonymised manner. Diagnostics were already finalised by the time of study.

### p16 staining

All cases were stained with an antibody against p16. Briefly, freshly cut 1 µm thick paraffin sections were stained using CINtec® (Roche, Basel, Switzerland) according to manufacturers’ instructions. Immunohistochemistry was performed using the DAKO FLEX-Envision Kit (Agilent, Santa Clara, CA, US) and the fully automated DAKO Omnis staining system (Agilent, Santa Clara, CA, US) according to manufacturer´s instructions. Epitope retrieval was generated at pH6 and 97 °C. Epitope staining was applied for 30 min. Epitope visualization was done by DAKO EnVision™ FLEX DAB + Substrate Chromogen System (Agilent, Santa Clara, CA, US). DAKO haematoxylin solution (Agilent, Santa Clara, CA, US) was used for nuclear counterstaining. Positive staining was noted in the case of block like positive staining. Mosaic staining pattern was noted as negative.

### DNA extraction

Tumor and non-tumor samples were macrodissected from 10 µm thick paraffin sections that were freshly cut. DNA extraction was performed applying the Maxwell 16 FFPE tissue LEV DNA purification kit (Promega, Madison, WI, USA). Quantification was performed using Quantus Fluorometer (Promega, Madison, WI). DNA quality was assessed by fragment analysis (ABI Genetic Analyzer, Thermo Fisher). HPV subtypes were analysed by Sanger sequencing and compared to LCD-Array technique (Chipron, Berlin, Germany).

### Sanger sequencing

Nested polymerase chain reaction (PCR) using GP5+/6+ and MY09/11 primers, commonly used for HPV detection, were tested on a subset of probes as described previously [10]. Primer sequences were: (5′–3′) MY9: CGTCCMARRGGAWACTGATC, MY 11 GCMCAGGGWCTATAAYAATGG, GP5 + TTTGTTACTGTGGTAGATACYAC, and GP6 + GAAAAATAAACTGTAAATCATATTC [10]. After PCR, a gel was run to test for products and correct sizes. In case a product was shown, it was subject to Sanger sequencing with My09/11 primers as described [10] on 3130xl Genetic Analyzer (ThermoFisher Scientific, Darmstadt, Germany) running the 3130 Series Data Collection Software v.3.0. PCR settings were the following: 95 °C for 2 min, and 39 times 95 °C for 1 min, 60 °C for 1 min and 72 °C for 1 min followed by 72 °C for 10 min. Termination was at 15 °C. Some probes were sent to Eurofins Genomics, Ebersberg, Germany for sequencing. The sequence of the forward and reverse reaction were subject to a Basic local Alignment Search Tool (BLAST) search [11]. One positive control containing HPV 16 was run with each sequencing test. Results were subject to evaluation via BLAST search in case of evaluable sequencing reults for forward and reverse primer. In case only one primer resulted in a sequence result it was declared negative.

### HPV testing with LCD-Array kit

**Fig. 1:**
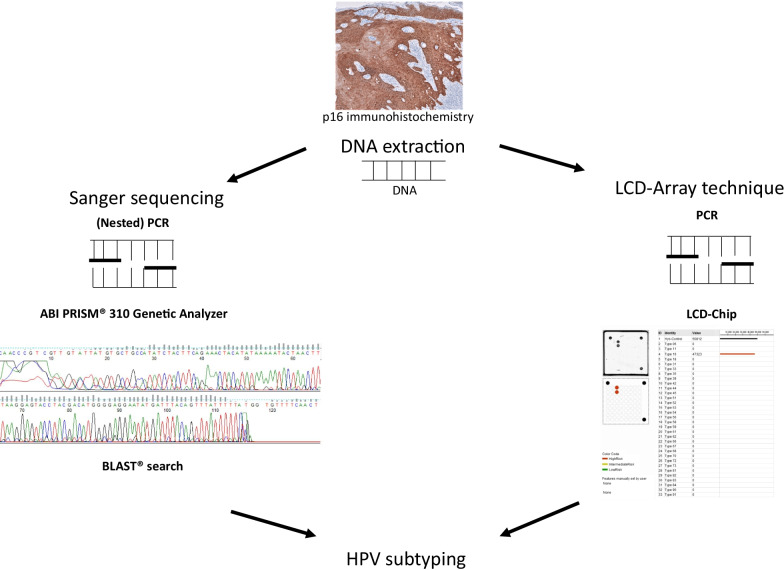
Positive staining for anti p16 antibody leads to the assumption of HPV infection. For further validation the prerequisite is DNA extraction to elucidate HPV infection status. Workflow for Sanger sequencing (left) and LCD-Array technique (right), simplified for visualisation means: Sanger sequencing demands for a PCR, which amplifies the sequence of interrogation with specific primer sets. Thereafter single strands are subject to capillary electrophoresis technology to retrieve the sequence of interest. After editing and proving the sequence output a BLAST® search can be applied to prove the sequence of the HPV subtype. For LCD-Array technique the workflow demands for a PCR reaction which leads to biotinylated PCR products. The product can be applied on an LCD-Chip. Image analysis is automated

All samples were re-analysed by applying HPV 3.5 LCD-Array Kit (LCD-Array, Chipron, Berlin, Germany), according to manufacturers’ instructions (https://www.chipron.com). PCR settings were the following: Start at 96 °C for 3 min, 42 times 94 °C for 1 min, 45 °C for 1 min and 30 s, 72 °C for 1 min and 30 s, finalized by 72 °C for 3 min and termination at 4 °C. The following HPV subtypes can be detected: 6, 11, 16, 18, 31, 33, 35, 39, 42, 44, 45, 51, 52, 53, 54, 56, 58, 59, 61, 62, 66, 67, 68, 70, 72, 73, 81, 82, 83, 84, 90 and 91. Tumor and non-tumor samples were tested in pairs. One positive and one negative control were taken per run. Figure 1 summarises the workflow and displays results.

### Data analysis

Data was tested for normal distribution and subject to tests according to their distribution using BiAS™ [12]. Distribution was tested using the Kolmogorov–Smirnov test. Further methods used were Spearman's rank correlation as well as Fisher’s exact test and contingency tables for sensitivity and specificity and Cohen’s d for calculation of effect strength and student’s t-test. Additionally Kaplan–Meier-estimator and Log-rank-test, Cox-Mantel and Peto-Pike were applied. Due to multiple testing, Bonferroni correction was applied.

## Results

### Tumor characteristics and growth pattern

The cohort of 70 penile neoplasias (62 invasive squamous cell carcinomas, 6 carcinomas in situ, one case of basal cell carcinoma, one case of extramammary Paget disease, focally invasive) was investigated. Two samples inherited either metastasis or central biopsy probe. All tumor and non-tumor specimens were screened, wherever applicable, for p16 and HPV status (in total 139 probes, since in one case only a metastasis could be evaluated).

Most of the invasive squamous cell carcinomas were stage pT1a and pT2. Six cases displayed Carcinoma in situ (6/70, 9%) and two cases (2/70, 3%) consisted of extramammary Paget disease, focally invasive and basal cell carcinoma.

In our cohort we detected exceedingly more squamous cell carcinomas, usual type (54/60, 90%), then squamous cell carcinomas, basaloid type (6/60, 10%). In many cases lymph nodes were not removed (pNX; 38/60, 63%). Most cases displayed no (lympho-)vascular invasion (L0, 47/60, 78%, V0, 54/60, 90%) and no perineural invasion (Pn0, 53/60, 88%). In most cases tumor grading was determined as G2 (39/60, 65%) on a scale G1–G3 (see Table 1 for a summary of tumor characteristics).

### P16 immunohistochemistry

In 70 samples we tested tumor and non-tumor for HPV and p16 status. One probe harboured only metastasis. In one sample p16 staining of non-tumor material could not be evaluated due to technical reasons (therefore n = 138). In total block like p16 positivity was detected in 39/138 (28%) evaluable samples.

Invasive SCCs (n = 62) were p16 positive in 32 cases (32/62, 52%). Carcinoma in situ were positive in 4/6 cases (67%). Extramammary Paget disease, focally invasive and basal cell carcinoma resulted negative for p16 staining.

For non-neoplastic epithelium 68 (68/69, 99%) cases were evaluable. Peritumoral epithelium was positive for p16 immunohistochemistry in 3 cases (3/68, 4%). All stainings are displayed in Additional file 1: Fig. 1 and Additional file 2: Fig. 2.

### Sanger sequencing

Overall, 137 samples consisting of tumor and non-tumor probes could be evaluated (137/139, 99%) by Sanger sequencing. HPV infection was detected in 21 samples (21/137, 15%).

61 of 62 (98%) invasive squamous cell carcinomas were evaluable via Sanger sequencing. 16/61 (26%) invasive carcinomas were positive for HPV infection: 13 cases showed positivity for HPV 16 (13/61, 21%). The remaining 3 cases harboured 2 co-infections HPV 16 and 33 (2/61, 3%) and one HPV 33 infection (1/61, 2%).

For carcinoma in situ (n = 6) all cases were evaluable (6/6, 100%). 1 case showed HPV infection (1/6, 17%) with HPV 16. The remaining 5 cases were negative for HPV infection (5/6, 83%).

Extramammary Paget disease, focally invasive and basal cell carcinoma were negative for HPV infection by Sanger sequencing.

Matching non-neoplastic epithelium was evaluable in 68/69 (99%) cases. 4/68 (6%) cases harboured HPV infection by HPV 16.

### HPV status via LCD-Array

In total 134/139 (96%) samples showed sufficient material for LCD-Array analysis. Of these HPV infection was detected in 58 probes (58/134, 43%).

**Fig. 2:**
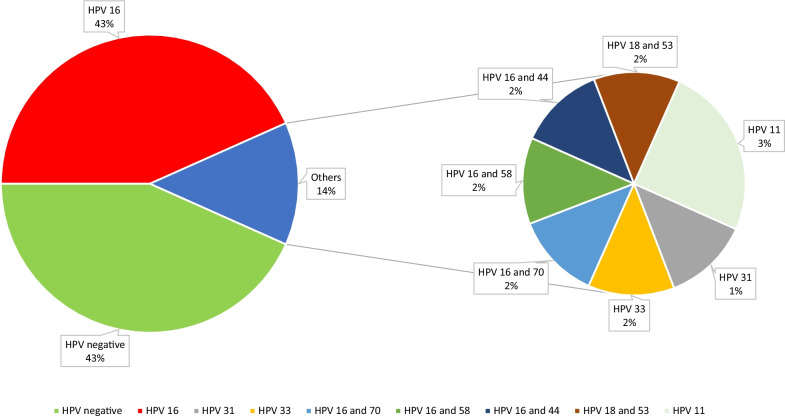
HPV status in invasive squamous cell carcinomas via LCD-Array technique

With LCD-Array technique, 60/62 (97%) invasive carcinomas were evaluable. 34/60 (57%) probes tested were positive for HPV infection. Among these, HPV 16 was the most frequent: 26/60 (43%). Eight remaining cases harboured infections by HPV 11 (2/60, 3%), HPV 31 (1/60, 2%), HPV 33 (1/60, 2%) and 4 cases showed co-infections of HPV 16 and 44, HPV 16 and 58, HPV 16 and 70 as well as HPV 18 and 53 (4/60, 7%). The results are summarised in Fig. 2.

Carcinoma in situ (n = 6) were tested positive for HPV infection in 100% of cases (6/6). 5/6 (83%) showed HPV 16 mono-infection. One case showed co-infection with HPV 16 and 33 (1/6, 17%).

Extramammary Paget disease, focally invasive and Basal cell carcinoma were both negative for HPV infection via LCD-Array technique.

For non-neoplastic epithelium, 66/69 (96%) were evaluable. 18/66 (27%) showed infection by HPV: HPV 16 was proven in 16/66 (24%) of cases. The remaining two cases harboured an infection by HPV 11 and a co-infection of HPV 16 and 33 (each 1/66, 2%).

Results for p16, Sanger sequencing and LCD-Array technique are summarised in Table 2.

**Table 2:** Results for p16 staining, Sanger sequencing and LCD-Array technique for squamous cell carcinomas, carcinoma in situ (Cais) and non-tumor tissue as well as overall

Sample	p16		Sanger sequencing		LCD-Array technique	
	positive (%)	negative (%)	positive (%)	negative (%)	positive (%)	negative (%)
Squamous cell carcinomas (n = 62)	32/62 (52%)	30/62 (48%)	16/61 (26%)	45/61 (74%)	34/60 (57%)	26/60 (43%)
Cais (n = 6)	4/6 (67%)	2/6 (33%)	1/6 (17%)	5/6 (83%)	6/6 (100%)	0 (0%)
non-tumor tissue (n = 69)	3/68 (4%)	65/68 (96%)	4/68 (6%)	64/68 (94%)	18/66 (27%)	48/66 (73%)
Overall (n = 139)	39/138 (28%)	99/138 (72%)	21/137 (15%)	116/137 (85%)	58/134 (43%)	76/134 (57%)

Extramammary Paget disease, focally invasive and basal cell carcinoma were negative for p16 staining, and showed no HPV infection via Sanger sequencing and LCD-Array technique

### Quality assurance (reference pathological assessment)

Five cases with positive results in peritumoral epithelium via LCD-Array technique were additionally investigated at another lab (https://www.aid-diagnostika.com/) by strip hybridization (AID Diagnostica) with 100% concordance.

LCD-Array technique detects HPV infection status directly in comparison to p16 testing and results in testing of multiple viruses in one sample. Therefore, we chose LCD-Array data as a gold standard for our statistical analysis. HPV status was based on LCD-Array data.

### Sensitivity and specificity

**Fig. 3:**
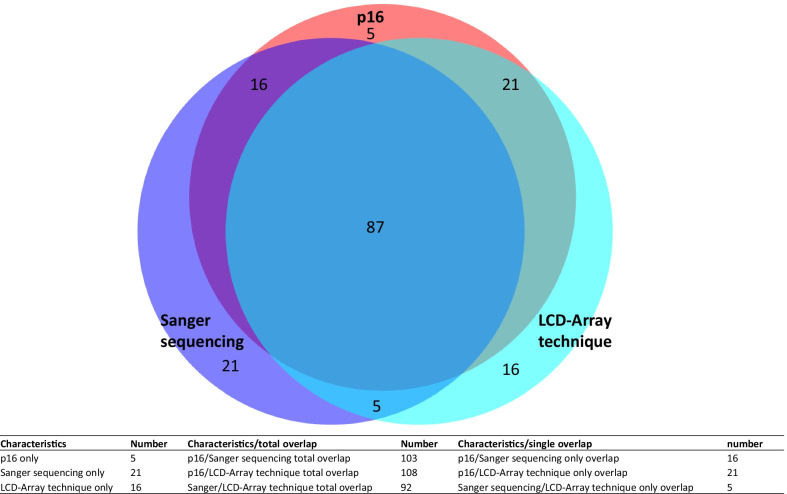
Venn diagram displaying results for p16, Sanger sequencing and LCD-Array technique for all probes with results for all techniques applied (n = 129). The table beneath presents number of overlaps between techniques

LCD-Array technique was taken as gold standard. For a direct comparison of all three techniques (Fig. 3, [13]) results were simplified as positive, negative and not evaluable. For p16, a positive block-like staining result was taken as positive and for sequencing techniques an evaluable measurement was taken as a result. For n = 131 samples data for all three techniques (p16, Sanger sequencing and LCD-Array technique) were applicable. Two cases revealed HPV 11 (low risk) and therefore negative p16 staining. These two cases were also not considered in direct comparison of all three techniques (n = 129).

The total overlap between all three techniques was 87 samples of 129 (67%). Overlap means that a positive p16 staining results in a HPV subtype determination via Sanger sequencing and LCD-Array and vice versa: negative p16 staining should lead to negative HPV proof by the other techniques.

The overlap between p16 and Sanger sequencing was 103/129 (80%) cases, the overlap between p16 and LCD-Array technique was 108/129 (84%) cases, and between Sanger sequencing and LCD-Array technique was 92/129 (71%) cases.

LCD-Array technique taken as gold standard lead to a sensitivity of 63% and a specificity of 100% for p16 staining, respectively. Prevalence was 43%. The rate of false positives was 0%, the rate of false negatives was 38%. Positive predictive value was 100%, negative predictive value was 78%.

Taking LCD-Array technique as gold standard Sanger sequencing had a sensitivity of 34% and a specificity of 100%. Prevalence was 43%. The rate of false positives was 0%, the rate of false negatives was 66%. Positive predictive value was 100% and the negative predictive value was 66%.

### Squamous cell carcinomas

For squamous cell carcinomas n = 57 samples contained measurements for all three techniques. The total overlap between all three techniques in squamous cell carcinomas was 39 (39/57, 68%).

**Fig. 4:**
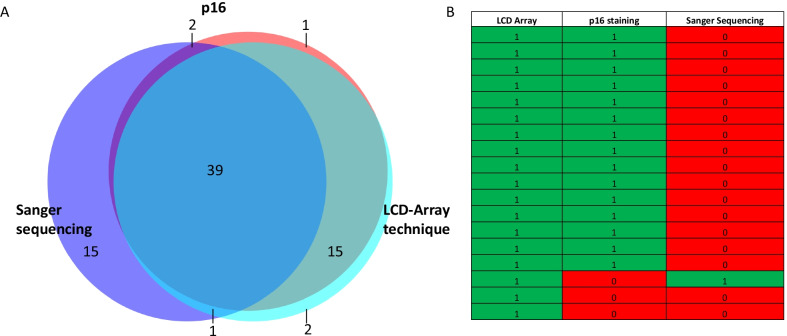
**A** Venn diagram displaying overlaps and controversies of p16, Sanger sequencing and LCD-Array technique in invasive squamous cell carcinomas of the penis. **B** Detailed report of the mismatches between all p16, Sanger sequencing and LCD-Array technique

Concordant and discrepant results in squamous cell carcinomas are displayed in Fig. 4. Sanger sequencing and LCD-Array technique showed overlapping negative results. For p16 staining 3 stains were negative but HPV 16 was shown via LCD-Array technique.

Looking at squamous cell carcinomas per se and taking LCD-Array as the gold standard, the prevalence for p16 positivity was 56%, sensitivity was 91%, specificity was 100%. The rate of false positives was 0%, the rate of false negatives was 9%. The positive predictive value of p16 staining was 100% and the negative predictive value was 89%.

Comparing Sanger sequencing and LCD-Array technique, taking LCD-Array technique as the gold standard, prevalence was 56%, sensitivity was 47%, specificity was 100%. The rate of false positives was 0% and rate of false negatives was 53%. The positive predictive value was 100% and the negative predictive value was 60%.

### HPV status via LCD-Array technique with regard to tumor characteristics and survival

HPV status was related to tumor characteristics. Fisher’s exact test showed a tendency towards an association between HPV status and tumor stage for LCD-Array technique (p = 0.027; Table 3; not significant after Bonferroni correction). Carcinoma in situ showed HPV infection in 100% of the cases (6/6) with six of six cases revealing HPV 16 infection and one of these cases with a co-infection 16 and 33. No HPV infection was detected in the basal cell cancer (0/1) and extramammary Paget disease, focally invasive (0/1). Bonferroni correction of p-values based on multiple tests showed no significant association between HPV status and tumor characteristics (Table 3). For comparison, the results are also displayed in relation to HPV status via p16 immunohistochemistry and Sanger sequencing in Additional file 4: Table 1a and 1b: For p16 status a statistical significant association between nodal stage (p = 0.04) and grading (p = 0.023) was shown. For Sanger sequencing a statistical significant association was shown regarding nodal stage (p = 0.01). The p-values displayed are not significant after Bonferroni correction. There was no significant difference regarding overall survival in this cohort with all three techniques as displayed in Additional file 3: Fig. 3.

**Table 3:** HPV status by LCD-Array technique with regard to tumor characteristics in evalulable cases

Characteristics	HPV high risk negative (n)	HPV high risk positive (n)	p***
pTX	2	0	0.027
pT1a	8	14	
pT1b	2	5	
pT2	15	7	
pT3	0	3	
pT4	0	2	
pNX	14	22	0.336
pN0	8	5	
pN1, pN2, pN3	5	4	
LX	1	0	0.743
L0	21	24	
L1	5	7	
VX	1	0	0.493
V0	23	29	
V1	3	2	
PnX	1	0	0.393
Pn0	22	29	
Pn1	4	2	
G1	9	3	0.087
G2	14	23	
G3	4	5	
usual morphology	25	27	0.675
basaloid morphology	2	4	
infiltrative pattern	16	20	0.788
pushing borders	11	11	

***Fisher–Freeman–Halton's exact contingency table-test. Bonferroni correction resulted in non-significant p-values

### Tumor size and infiltration depth

The mean tumor size was 30 mm overall (median: 22 mm; range 4–140 mm; n = 49 evaluable samples). The mean infiltration depth was 6 mm (median: 5 mm; range 1–24 mm; n = 49 evaluable samples). There was no significant association between tumor size and infiltration depth in relation to HPV status obtained via LCD-Array technique (Table 4).

**Table 4:** Influence of HPV status detected via LCD-Array on tumor size and infiltration depth

Tumor size	Infiltration depth					
	HPV neg	HPV pos	Cohen's effect size d with pooled standard deviation****	HPV neg	HPV positive	Cohen's effect size d with pooled standard deviation****
N	20	29	0.27	23	26	0.11
Mean (mm)	26	32	(Welch ‘s t-test: t = 1.01 (p = 0.32))	7	6	
Median (mm)	23	22		5	5	
STD DEV	18	29		6	5	
Min (mm)	7	4		1	1	
Max (mm)	85	140		24	17	

****Cohen: d = 0.2 small effect, d = 0.5 medium effect, d = 0.8 big effect

## Discussion

To establish the LCD-Array hybridisation technique in routine diagnostics for the HPV analysis of penile carcinomas, we examined penile carcinomas, penile carcinomas in situ und peritumoral epithelium expecting a variable rate of HPV infections.

As a surrogate for possible HPV infection in routine diagnostics, p16 immunohistochemistry is widely applied, for example in oropharyngeal carcinomas, where a positive staining will serve as a marker for clinical behaviour of the tumor [14]. In our cohort of invasive squamous cell carcinomas we found three cases without p16 staining but with a positive HPV test, when applying the LCD-Array technique. Varying expression patterns of p16 may be derived from certain reasons: Negative or partially positive p16 staining can, in case of infection, be explained by gene methylation, loss of the protein in tumor cells due to genetic instability or low risk HPV infection [15]. Therefore, molecular techniques can shed light on an increasing number of patients that would have been missed in cases of negative p16 staining and can be applied as additional techniques.

Sanger sequencing with established primers was performed on archived formalin fixed paraffin embedded material in a lab routinely using Sanger sequencing method. Sanger sequencing is a technique, which is well established and is called the “gold-standard” for the validation of newly developed sequencing methods, also in HPV testing [16].

The advantage of Sanger sequencing is the interrogation of a longer segment, whereas LCD-Array technique is limited to certain subtypes of HPV.

Technically, hands on time differs between Sanger sequencing and LCD-Array: The LCD-Array Chip can be finished within one working day, whereas Sanger sequencing takes approximately two working days. DNA input and costs do not differ significantly.

The evaluation of results is more time consuming via Sanger sequencing technique since the LCD-Array hybridisation method has a visual feedback mechanism. Data interpretation of Sanger sequencing results demands experience and is partly subjective. Figure 1 gives an example for a positive p16 staining, Sanger sequencing and LCD-Array result. Limit of detection varies depending on the mutation investigated [17]. Discrepancies between Sanger sequencing and LCD-Array hybridisation technique can partly be attributed to differing sensitivities and specificities: For HPV it is known that the viral load can vary and is not necessarily related to cancer development [18, 19]. Detection of co-infections via Sanger sequencing was inferior to LCD-Array technique, which is in line with a past study [16].

In our study, we applied FFPE material for HPV detection. FFPE material is a widely available material in pathologies. Due to fixation artefacts and storage time DNA yield can vary and can sometimes result in probe failure [20].

As of 2021 the Food and Drug Administration (FDA) approved several HPV testing methods: APTIMA HPV 16 18/45 Genotype Assay and APTIMA HPV Assay (Gen-Probe, Inc., San Diego, CA), Cervista HPV 16/18 and Cervista HPV HR and Genfind DNA Extraction Kit (Hologic, INC., Marlborough, MA), COBAS HPV Test and cobas HPV for use on the cobas 6800/8800 Systems (Roche Molecular Systems, Inc., Pleasanton, CA), Digene Hybrid Capture 2 High-Risk HPV DNA Test (QIAGEN GAITHERSBURG, INC, Germantown, MD) and BD ONCLARITY HPV ASSAY (BECTON, DICKINSON AND COMPANY, Sparks, MD) [21].

APTIMA HPV 16 18/45 Genotype Assay is indicated for the qualitative detection of E6/E7 viral messenger RNA (mRNA) of HPV types 16,18 and 45. It differentiates between HPV 16 and HPV 18 and/or HPV 45 [21]. APTIMA HPV ASSAY is an amplification test for the qualitative detection of E6/E7 (mRNA) to recognize HPV 16, 18, 31, 33, 35, 39, 45, 51, 52, 56, 58, 59, 66, 68. Discrimination of the HPV types is not intended [21]. Cervista HPV 16/18 qualitatively detects DNA from HPV type 16 and 18 by fluorescence signal [21]. Cervista HPV HR and Genfind DNA Extraction Kit is a qualitative test for the detection of 14 high risk HPV types (16, 18, 31, 33, 35, 39, 45, 51, 52, 56, 58, 59, 66, and 68) not discriminating the type [21]. COBAS HPV Test is PCR based and utilises nucleic acid hybridisation to detect 14 high risk HPV types, of which 16 and 18 are discriminated. The others include 31, 33, 35, 39, 45, 51, 52, 56, 58, 59, 66 and 68 [21]. The digene HC2 High-Risk HPV DNA Test detects 13 high-risk HPV types, which are 16, 18, 31, 33, 35, 39, 45, 51, 52, 56, 58, 59, 68 using full genome probes complementary to HPV DNA, specific antibodies, signal amplification, and chemiluminescent detection. It analyses HPV DNA high-risk groups [22]. BD Onclarity HPV Assay is PCR-based and uses nucleic acid hybridisation for the detection of 14 high risk HPV types discriminating 16, 18 and 45 and detecting 31, 33, 35, 39, 51, 52, 56, 58, 59, 66 and 68 [21].

A study comparing sensitivity and specificity of the above mentioned tests showed that all DNA/RNA-based tests, except the NorChip test, showed high sensitivity rates for high-grade lesions positive by cytology [23]. These tests apply to non-FFPE material and test for limited subtypes of HPV, as described above.

The LCD-Array’s performance was demonstrated by the HPV Laboratory Network (LabNet) in comparison to other tests with 100% proficient results [24]. The advantage of this test is its usability on FFPE material. Additionally, the aforementioned tests miss a certain amount of HPV infections. Those were all found by applying the LCD-Array technique in a recent study on gynaecological samples [25]. Studies comparing HPV genotyping methods described differences in HPV genotype detection depending on the technique used with concordance rates of minimum 77,6%, declining when only looking at FFPE material [26–28]. Therefore, taking into account that archived FFPE material was used in this study the concordance rate seen here could also be attributed to DNA degradation.

The cohort of Frankfurt penile carcinomas and carcinomas in situ was associated with HPV infection in 61% (40/66 evaluable samples; 6 Carcinoma in situ, 34 invasive carcinomas) of the cases as defined by LCD-Array technique, which is in line with other studies [2, 15] and higher than in a recent review [29]. Basal cell carcinoma was negative for HPV infection using PCR-based techniques and via p16 staining. Cases of p16 positive basal cell carcinomas were described in the literature [30]. Nevertheless, in our case, HPV subtyping for mucosal/genital high risk viral infection of the HPV was negative. Most likely the HPV infection was not detected by our techniques because the specific beta-family of HPV subtypes, mostly associated with these tumors [31], was not interrogated by our assays. For extramammary Paget disease, p16 expression is also reported in the literature. There is variable attribution to HPV infection status [32].

We were able to show HPV infection in peritumoral epithelium (18/66, 27%; see above). Positive measurement in non-neoplastic epithelium resulted in positive measurement in neoplastic epithelium. The question needs to be raised, why, despite HPV infection in both fractions, one resulted in tumor growth and the other did not. The HIM Trial postulates up to 37 HPV types in the genitals, of which ca. 5% progressed to a genital lesion [33]. It can be speculated, whether this might be due to transient HPV infections, which can be cleared by the immune system [34]. Furthermore the observations need to be questioned concerning their clinical impact: The pure measurement of HPV infection via molecular techniques in non-neoplastic tissue may account for a sensitive technique but could raise concerns in the management of the patient. Negative p16 stainings in non-neoplastic epithelium in case of positive measurements by molecular techniques should therefore, formally not be regarded as false negatives. For this reasen molecular techniques should be used in addition to p16 stainings for uncertain cases or maybe in the setting of clinical trials.

As expected, the most frequent type of HPV infection in squamous cell carcinomas resulted from HPV 16. That is in line with previous studies [35].

In the tumor cohort, we investigated squamous cell carcinomas of the usual type and basaloid subtype. Although the usual type is classified as non-HPV-related, according to the WHO classification, we found 30 samples with HPV infections among 54 evaluable tumor samples described as squamous cell carcinoma, usual type (56% positive samples), which is in line with a previous review [36]. We have found no significant association between HPV infection status and tumor characteristics according to TNM classification system as well as tumor size, overall survival and infiltration depth in our cohort. Differing prognosis for HPV positive and HPV negative cases have been reported in other cancer types, such as head and neck cancer [37]. Moreover, in our cohort a significant association between overall survival depending on the technique for HPV detection applied was not shown. Maybe this can partly be attributed to sample size.

Next generation sequencing (NGS) methods have been applied to tumor tissue and revealed subtypes not known before the advent of these techniques [38]. Additionally, investigations on viral integration into the genome, detected by next generation sequencing, lead to insights into tumor pathogenesis [39]. Due to HPV vaccination a shift in prevalence of HPV types can be expected which makes testing for numerous HPV types important [40].

HPV was also detected in lymph node metastases. There are approaches to detect circulating cell free HPV DNA as a measure for disease control in oropharyngeal cancer [41]. This technique may eventually be applicable to penile carcinomas and could be used as a non-invasive test for the prediction of high risk for systemic disease.

In the setting of the LCD-Array test 32 HPV types can be detected, which exceeds the types tested by the aforementioned tests. Currently it is not feasible to apply NGS methods to the large amount of samples observed in daily routine, as the costs exceed the possible compensation. This is why LCD-Array technique is a fast and reliable alternative for routine practice.

## Conclusions

p16 staining is in most cases sufficient for the detection of high risk HPV association of penile neoplasias. LCD-Array is a feasible, sensitive and specific, as well as cost efficient diagnostic tool for HPV testing of FFPE tissue of penile cancer which can be applied in routine testing in addition to p16 staining or in clinical trials. LCD-Array technique may serve as a helpful tool in the advent of other HPV subtypes emerging in the setting of HPV vaccinated patients. Readouts of molecular techniques must be interpreted with caution to not over interpret transient HPV infections in the setting of a positive HPV detection in non-neoplastic epithelium.

## Supplementary Information

**Additional file 1: Fig. 1.** Negative p16 stainings.

**Additional file 2: Fig. 2.** Positive p16 stainings.

**Additional file 3: Fig. 3.** **A** Kaplan–Meier-Estimator for p16 positive and negative staining for overall survival in the cohort of penile neoplasias (p = 0.43; Log-rank-test, Cox-Mantel and Peto-Pike). **B** Kaplan–Meier-Estimator for LCD-Array with regard to HPV high risk positive and HPV high risk negative cases for overall survival in the cohort of penile neoplasias (p = 0.59; Log-rank-test, Cox-Mantel and Peto-Pike). **C** Kaplan–Meier-Estimator for Sanger sequencing with regard to HPV high risk positive and HPV high risk negative cases for overall survival in the cohort of penile neoplasias (p = 0.62; Log-rank-test, Cox-Mantel and Peto-Pike).

**Additional file 4: Table 1a.** HPV status of invasive squamous cell carcinomas by p16 immunohistochemistry with regard to tumor characteristics in evaluable cases**.** Table 1b**. HPV status by Sanger sequencing technique with regard to tumor characteristics in evaluable cases**.

## Data Availability

The datasets used and/or analysed during the current study are available from the corresponding author on reasonable request.
