# Supplementary figures and images for: The routine use of LCD-Array hybridisation technique for HPV subtyping in the diagnosis of penile carcinoma compared to other methods
Source: BMC Urol. 2022 Jan 29;22:10. doi: 10.1186/s12894-022-00962-4 (PMC8801096; doi:10.1186/s12894-022-00962-4)

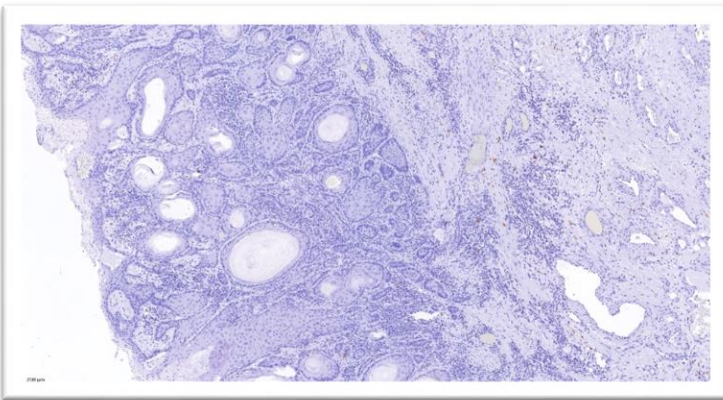

(1)

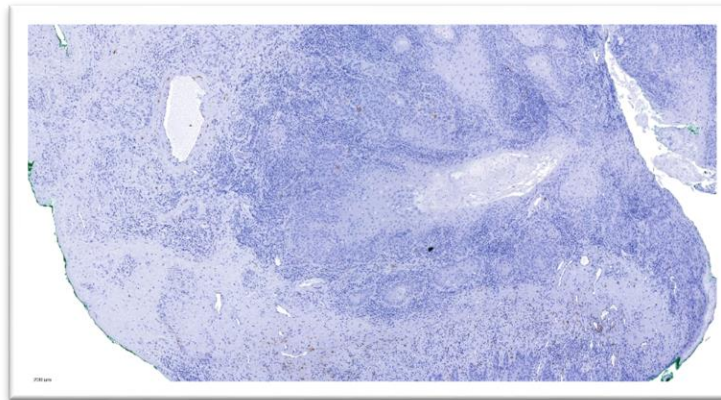

(2)

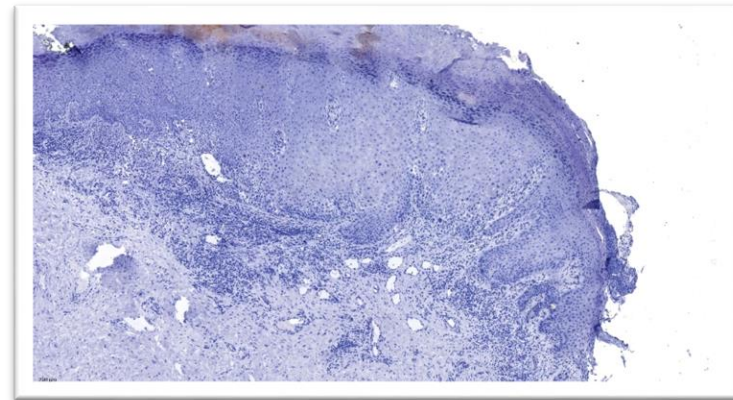

(3)

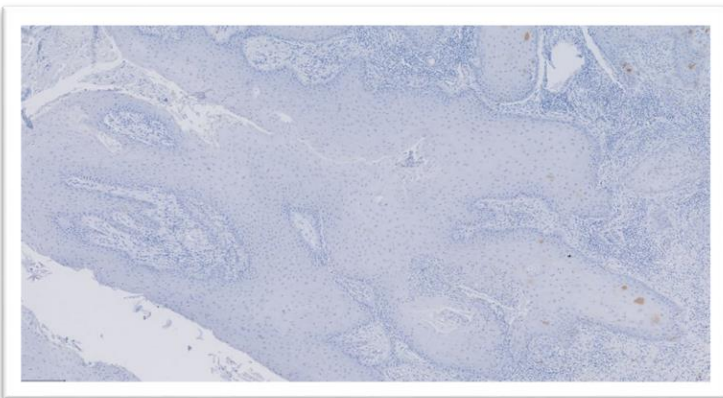

(4)

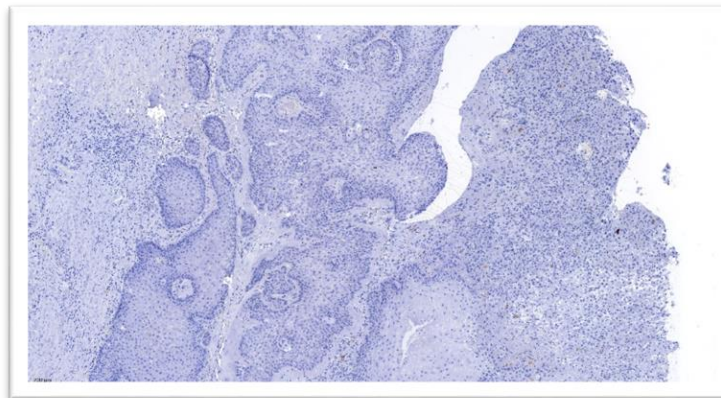

(5)

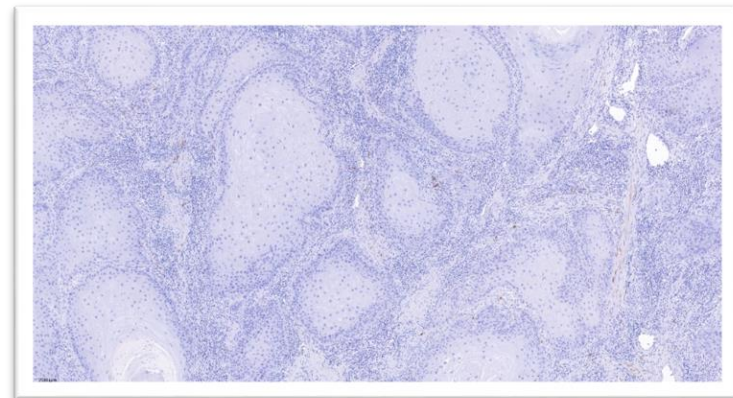

(6)

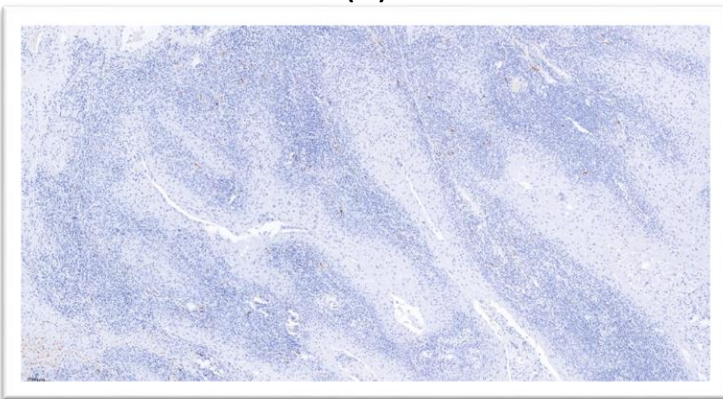

(9)

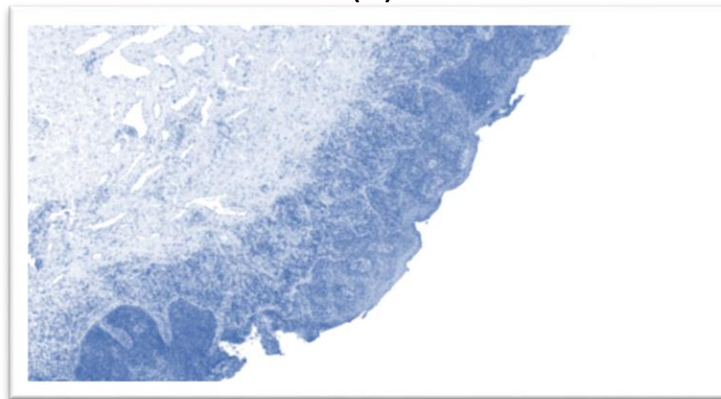

(11)

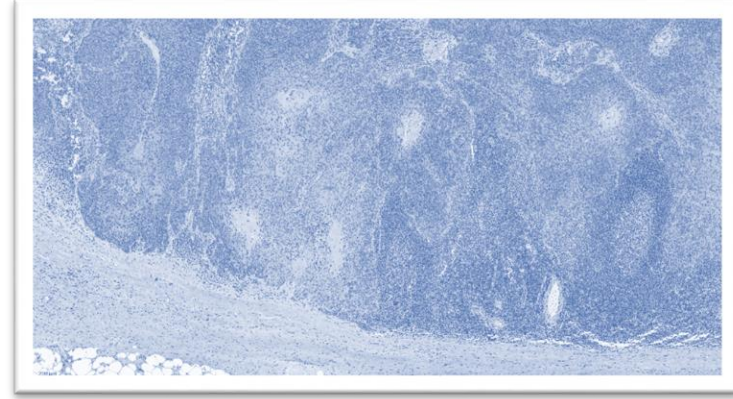

(12)

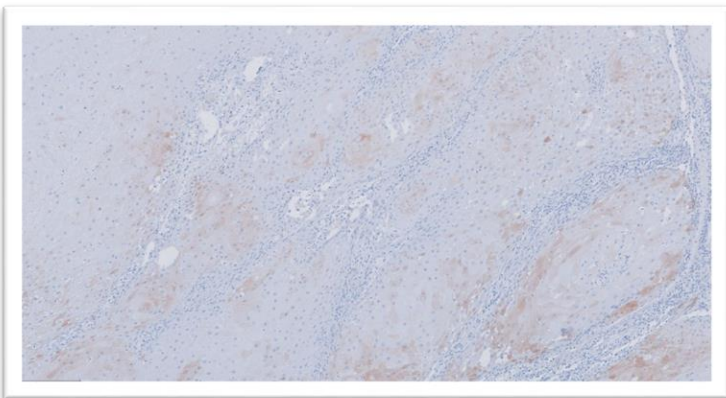

(14)

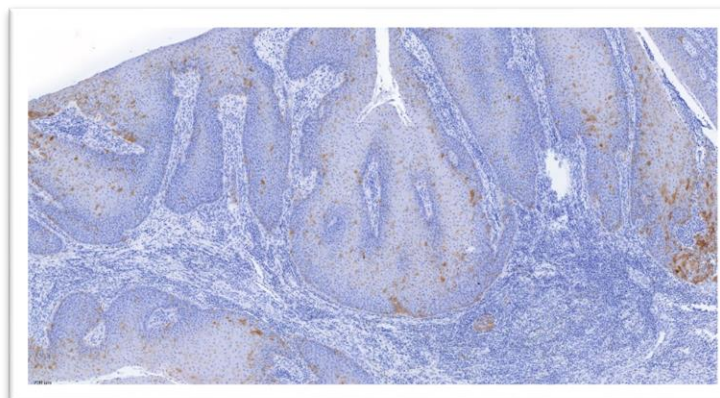

(17)

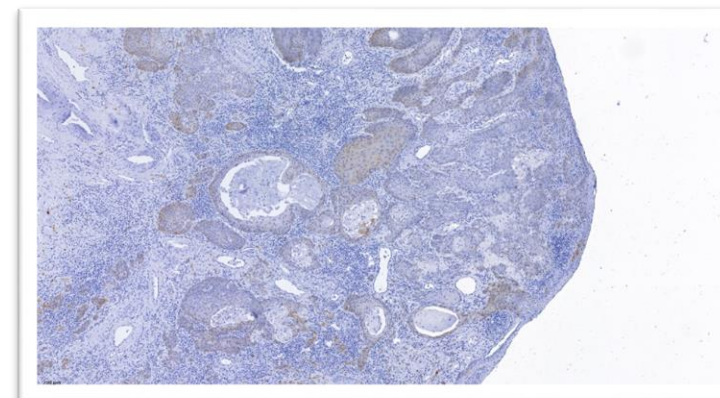

(21)

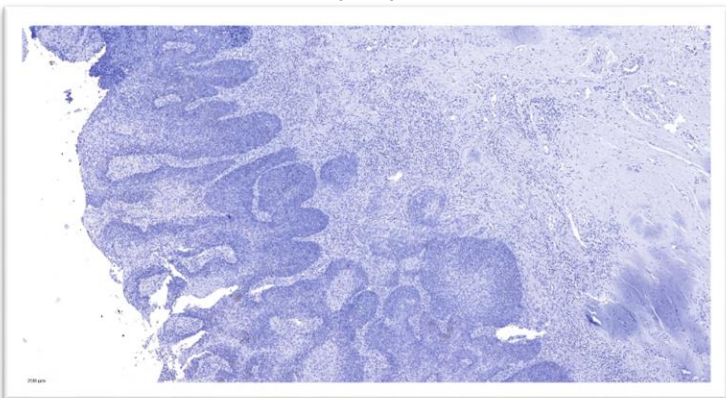

(26)

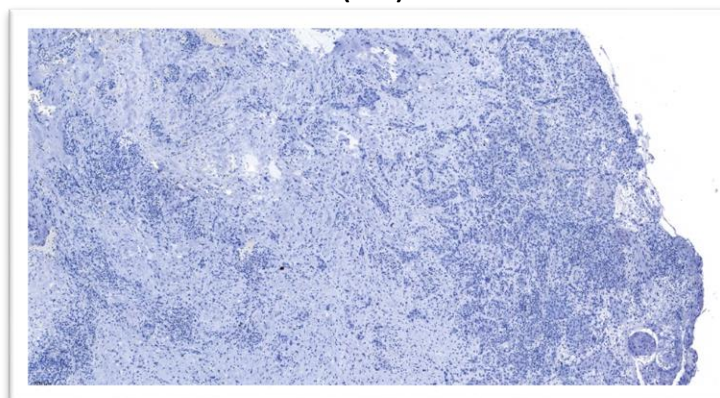

(28)

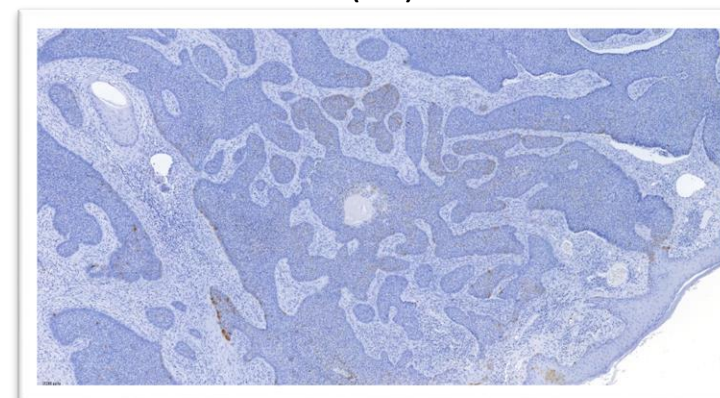

(33)

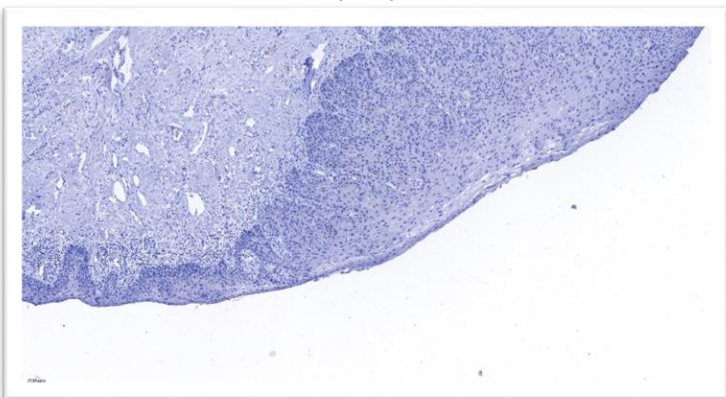

(34)

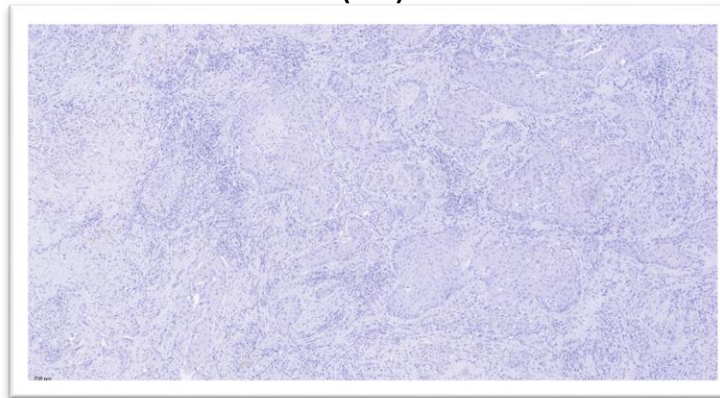

(35)

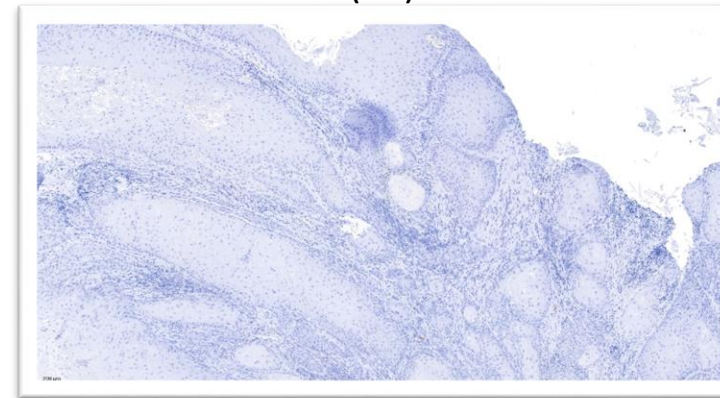

(36)

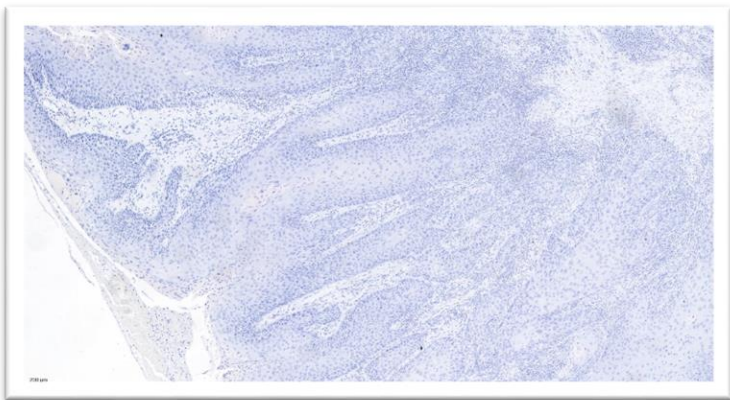

(38)

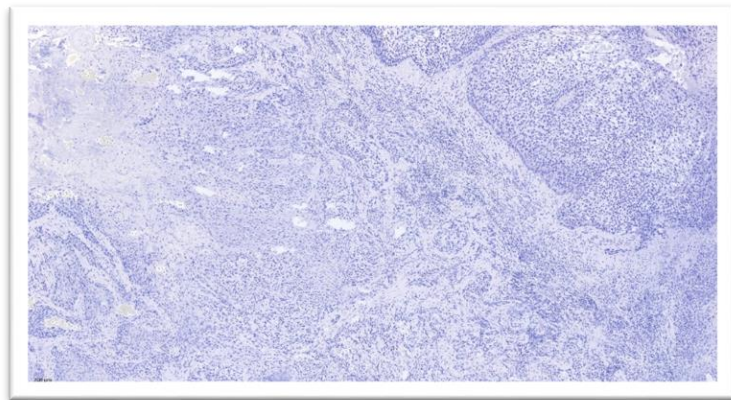

(43)

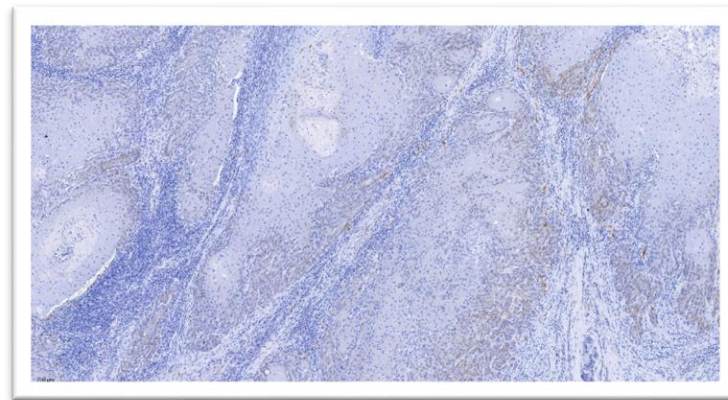

(44)

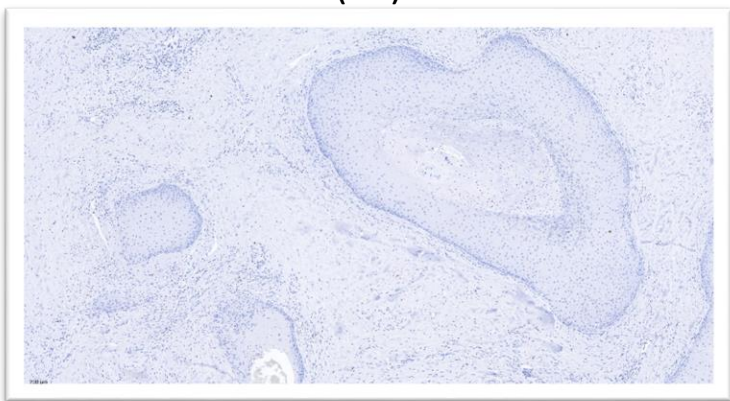

(45)

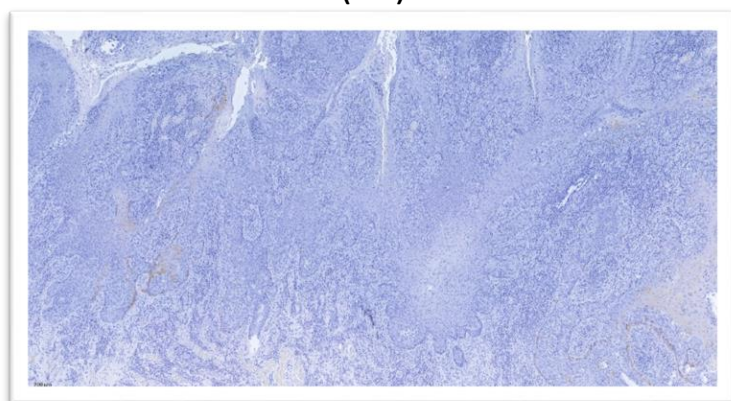

(47)

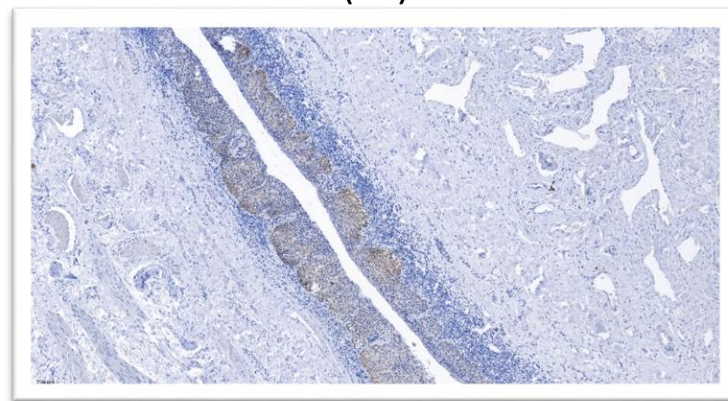

(48)

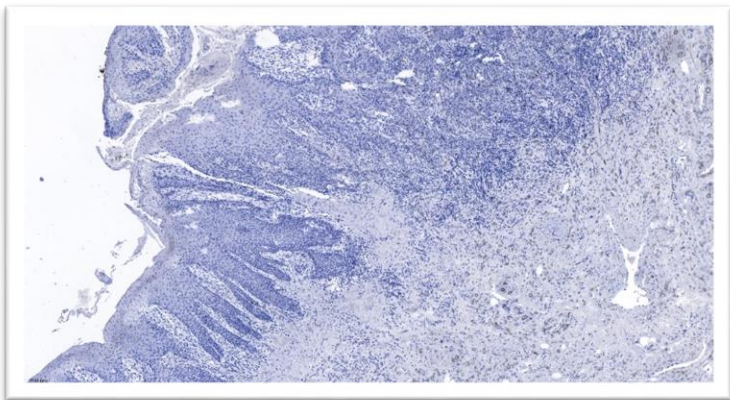

(50)

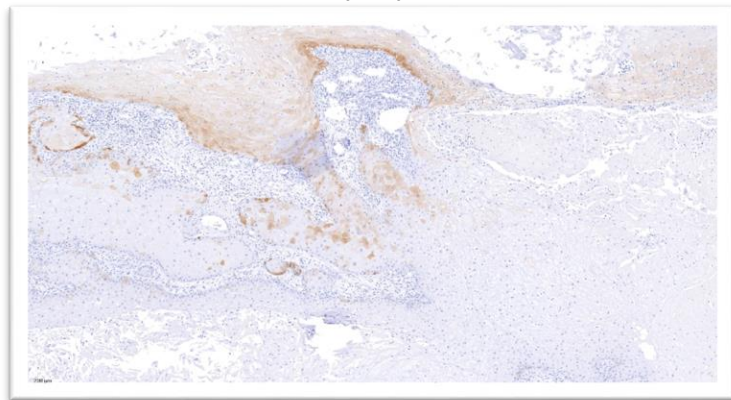

(51)

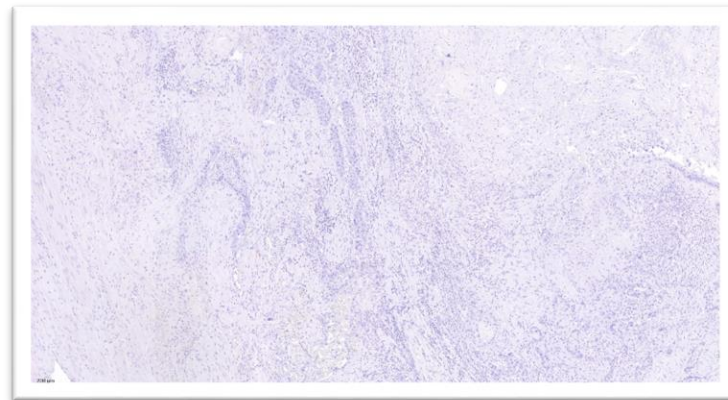

(53)

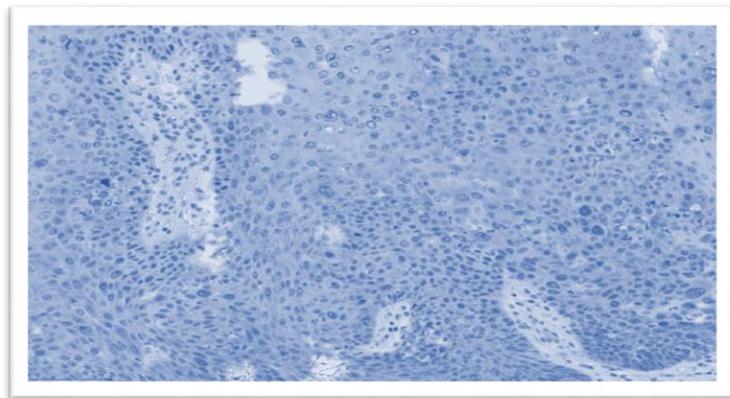

(58)

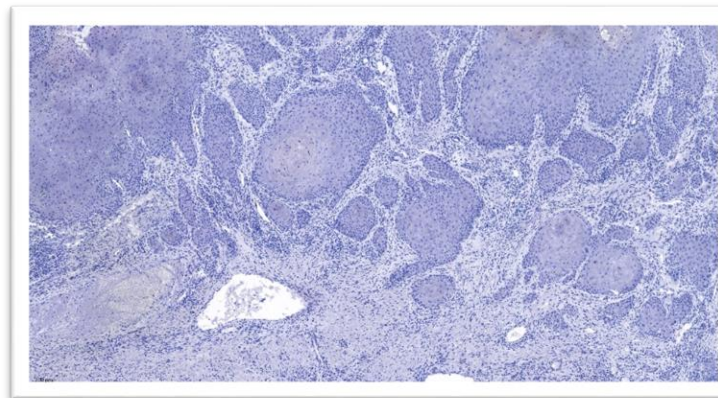

(59)

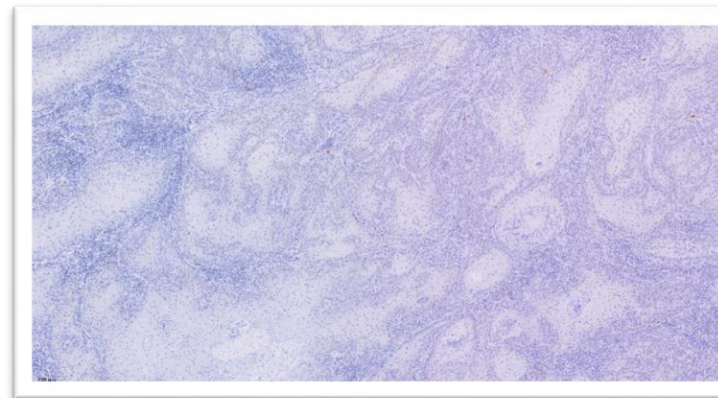

(61)

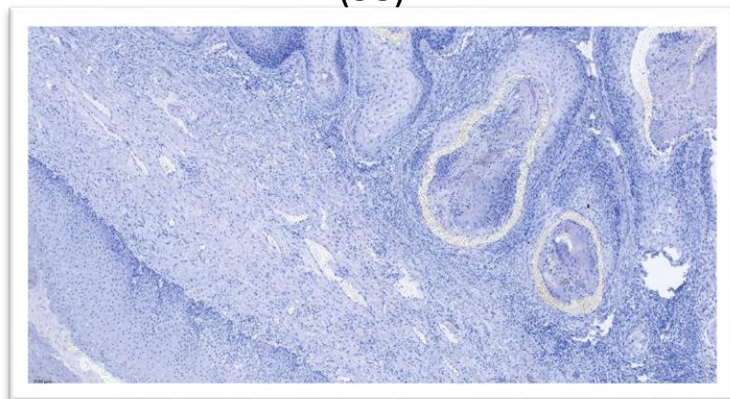

(62)

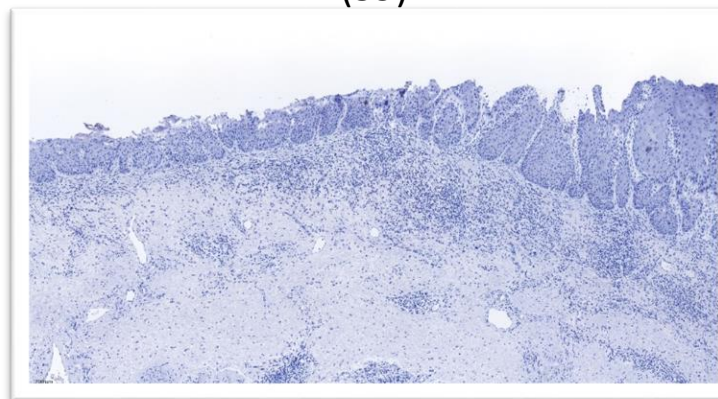

(64)

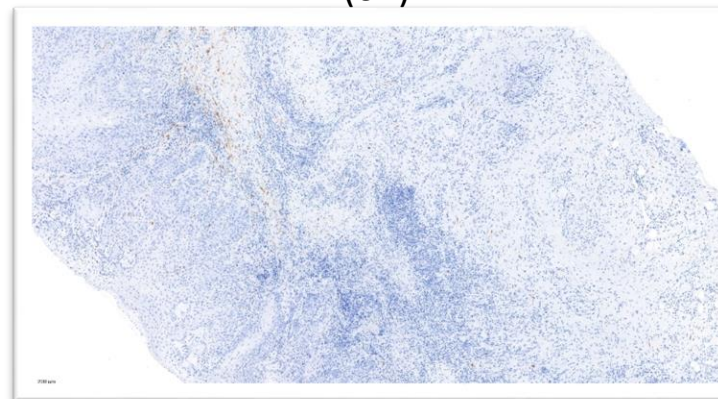

(67)

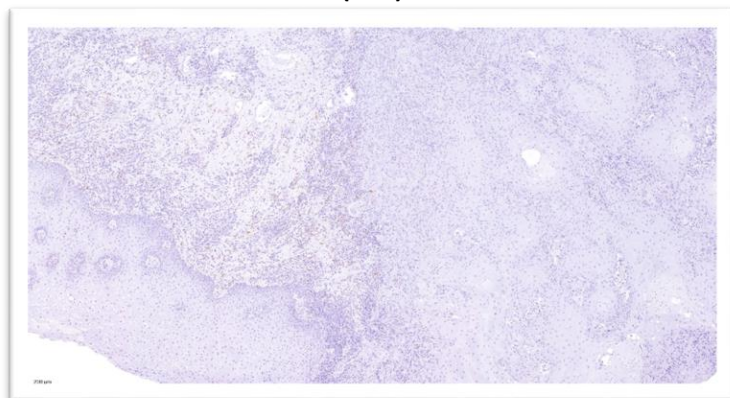

(69)

Supplementary Figure 1

Supplement: Supplementary file 1 — Additional file 1: Fig. 1. Negative p16 stainings. [file 12894_2022_962_MOESM1_ESM.pdf]

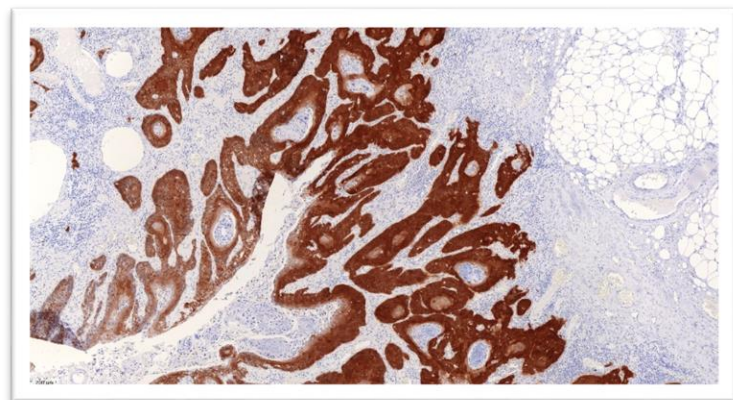

(7)

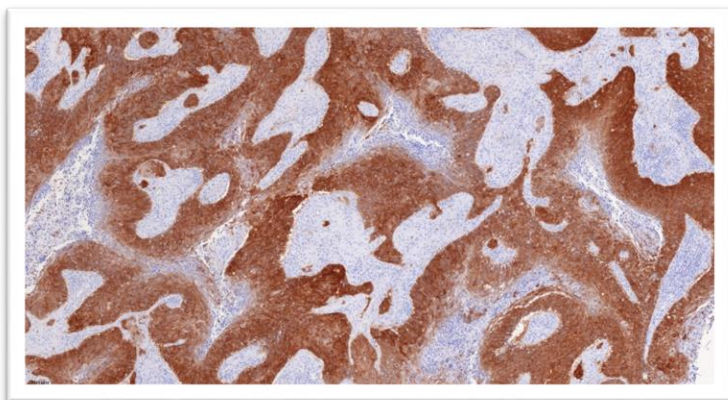

(8)

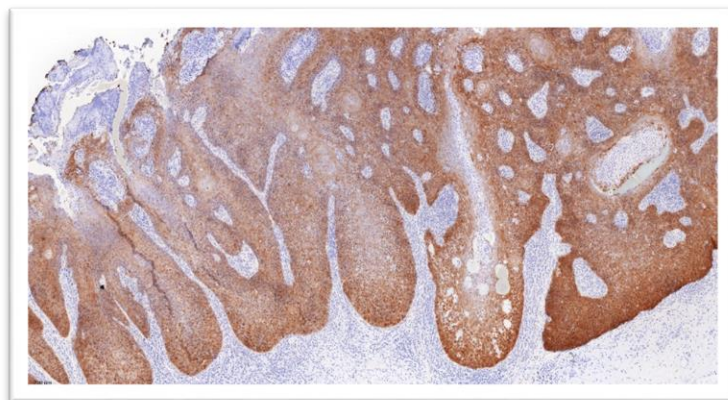

(10)

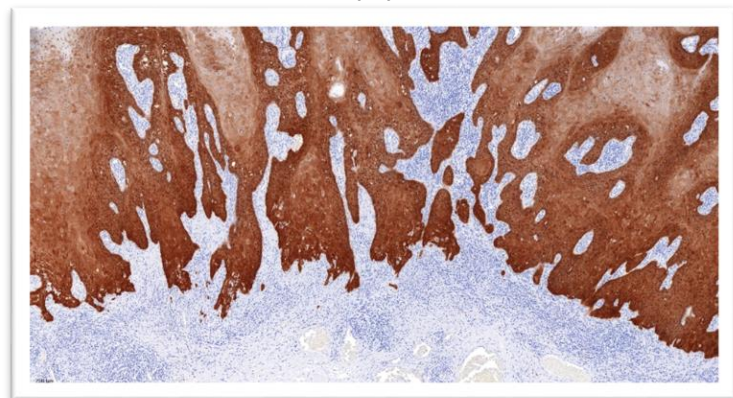

(13)

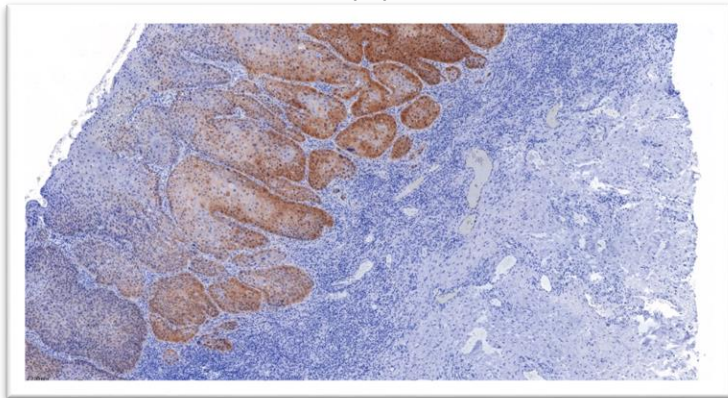

(15)

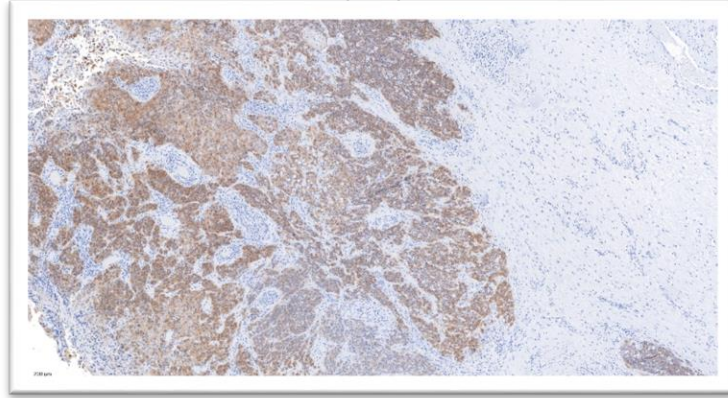

(16)

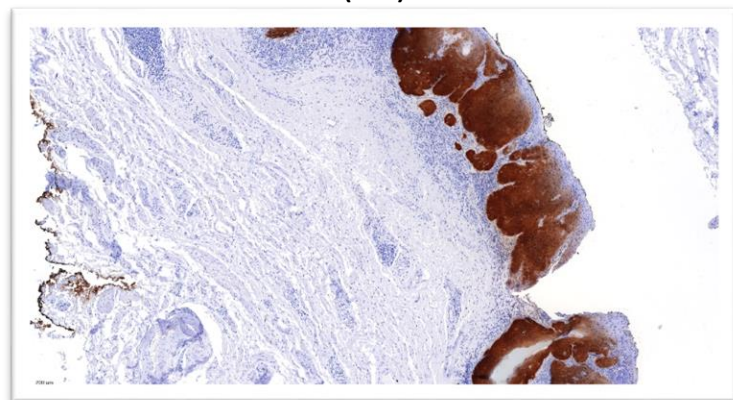

(18)

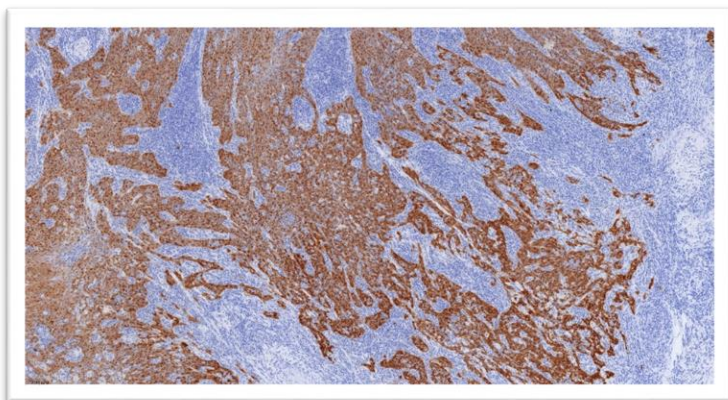

(19)

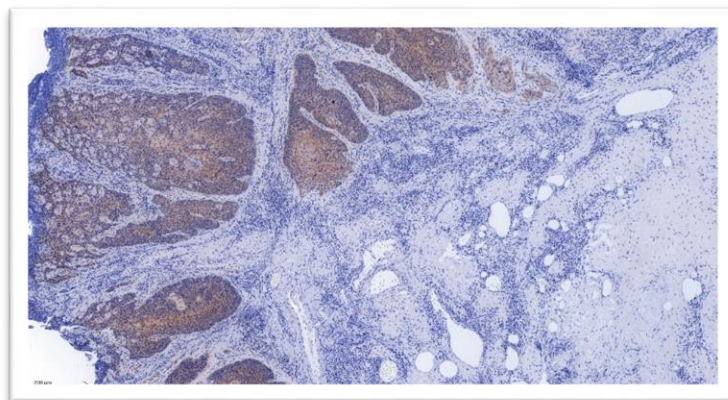

(20)

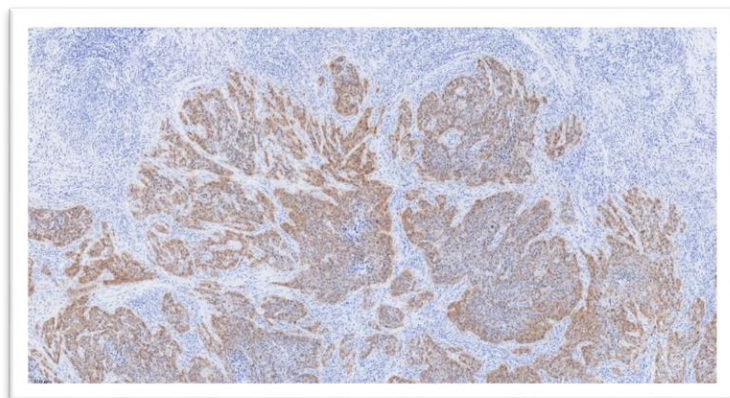

(22)

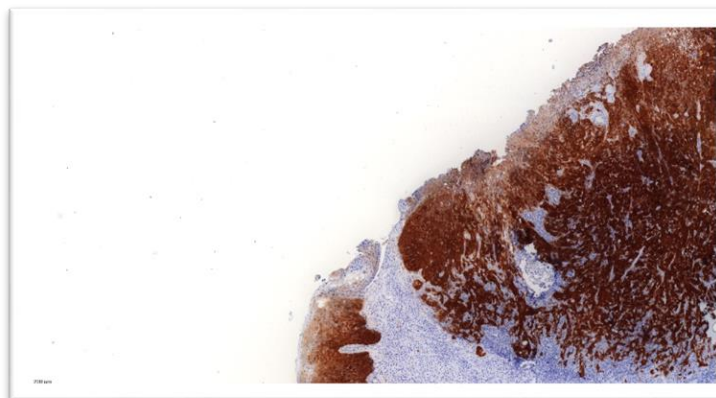

(23)

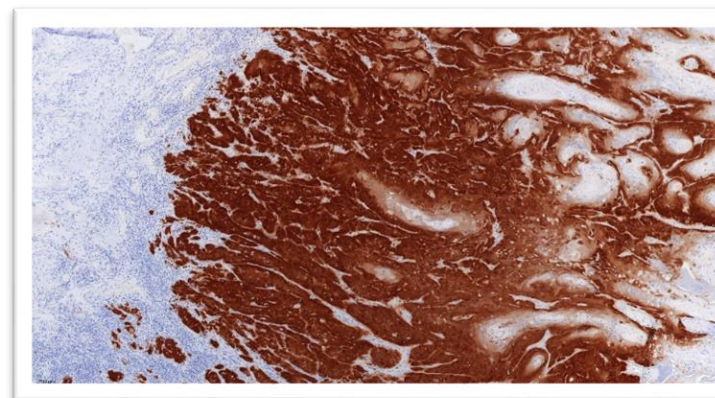

(24)

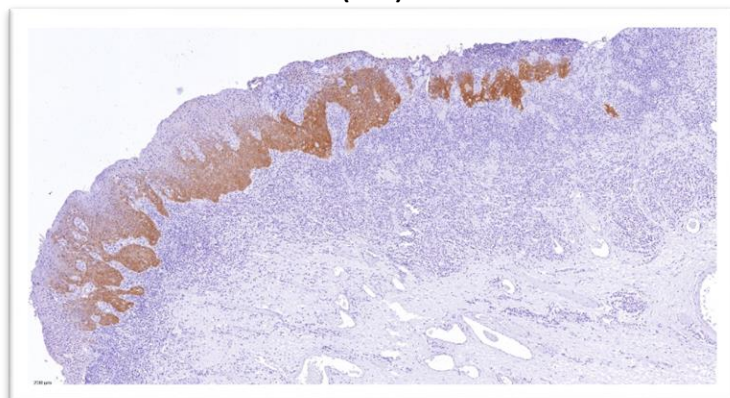

(25)

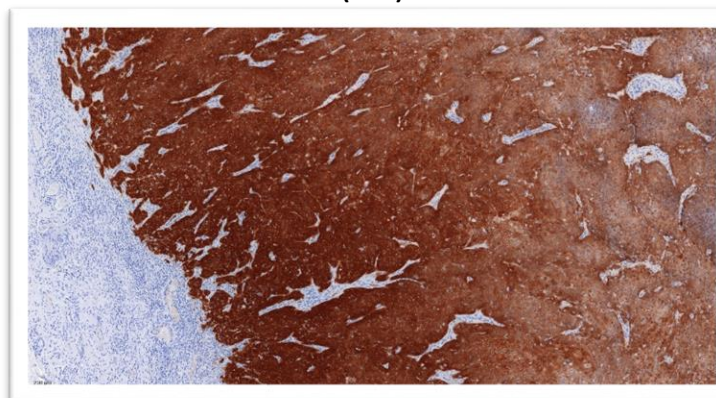

(27)

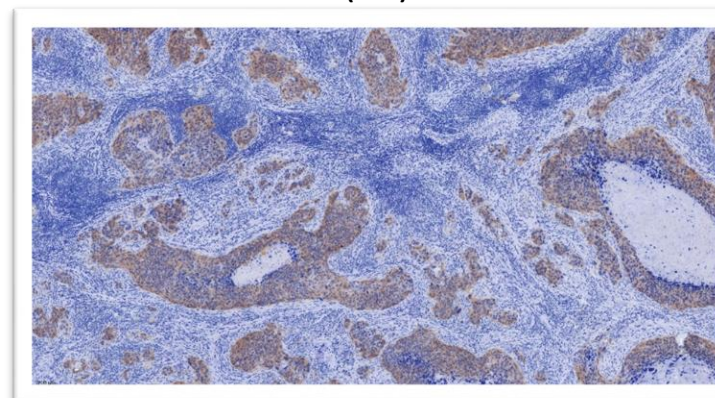

(29)

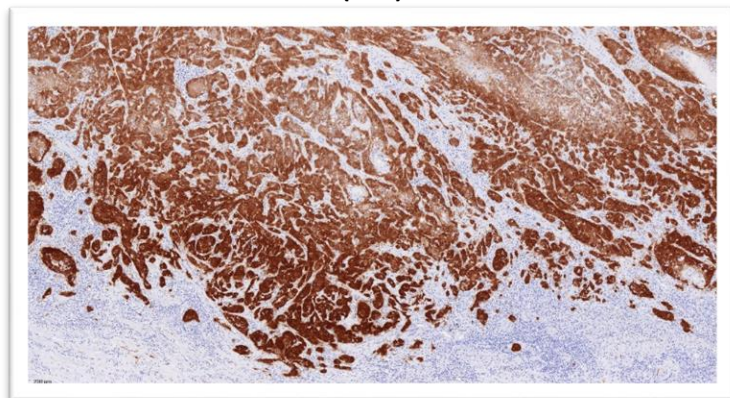

(30)

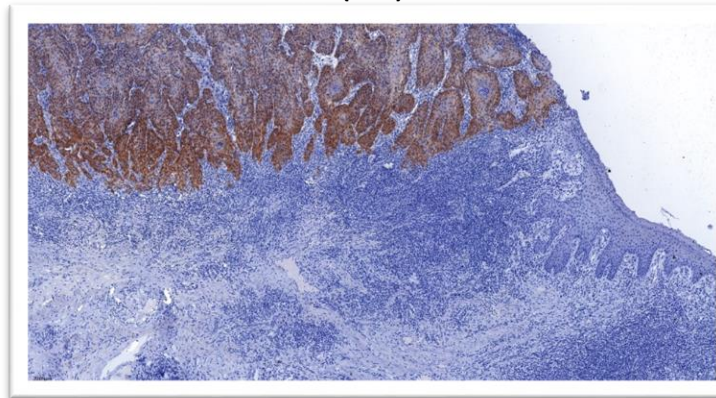

(31)

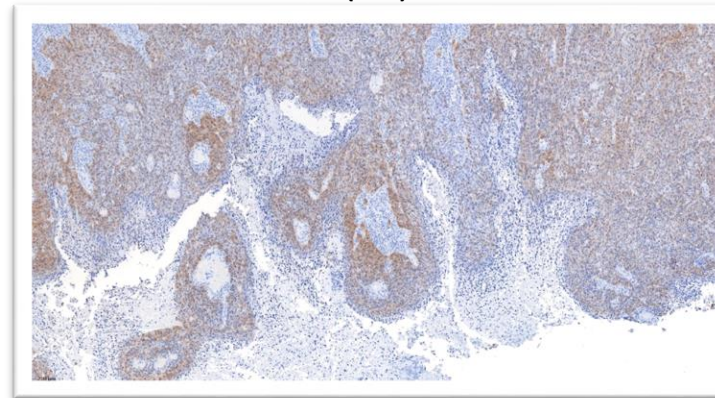

(32)

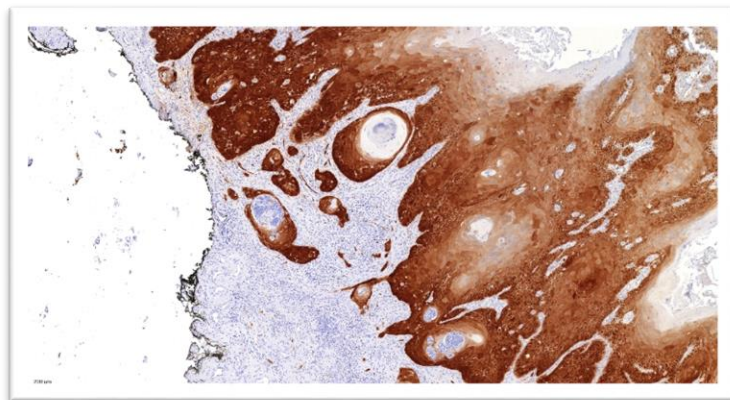

(37)

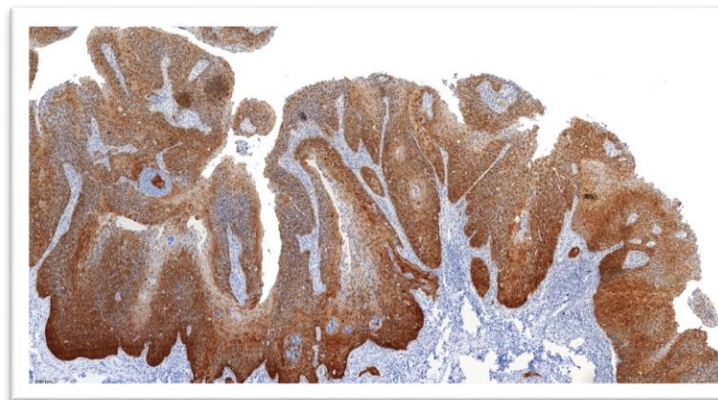

(39)

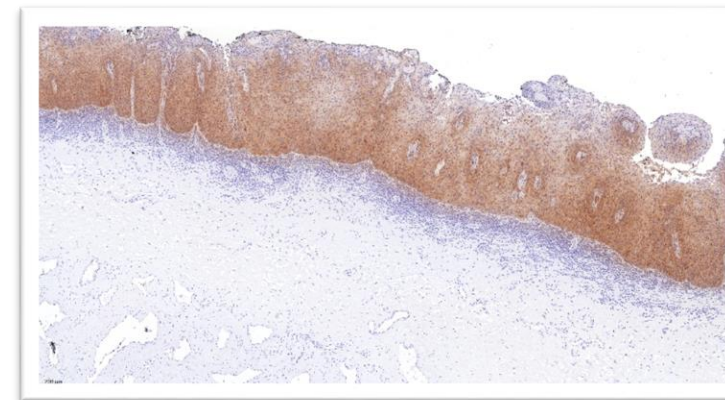

(40)

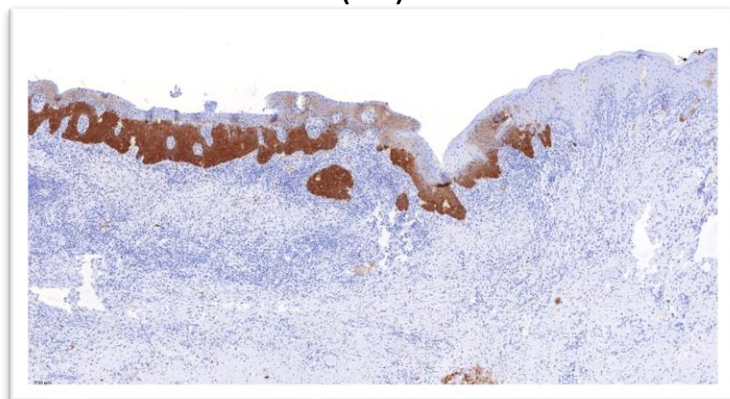

(41)

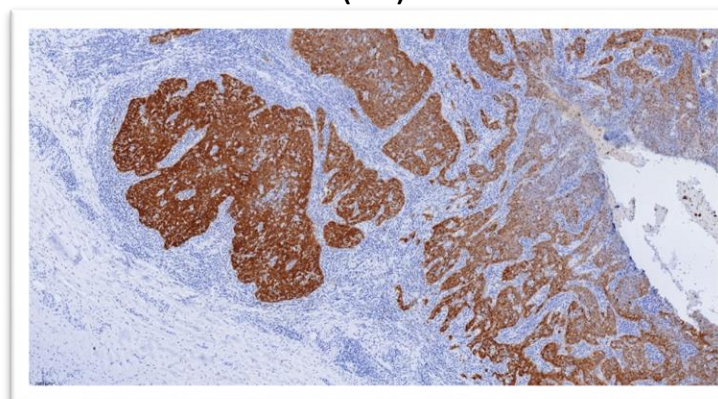

(42)

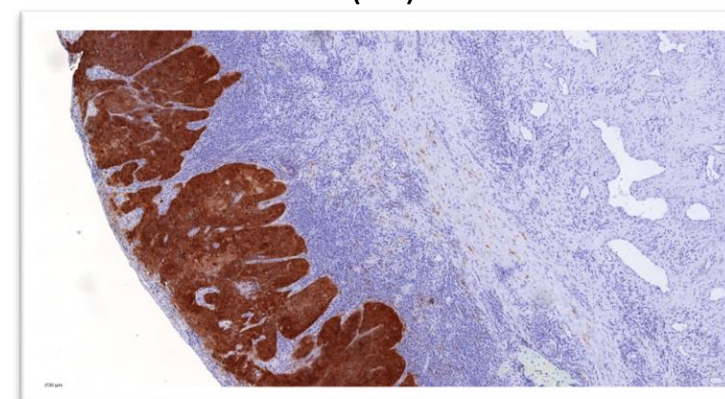

(46)

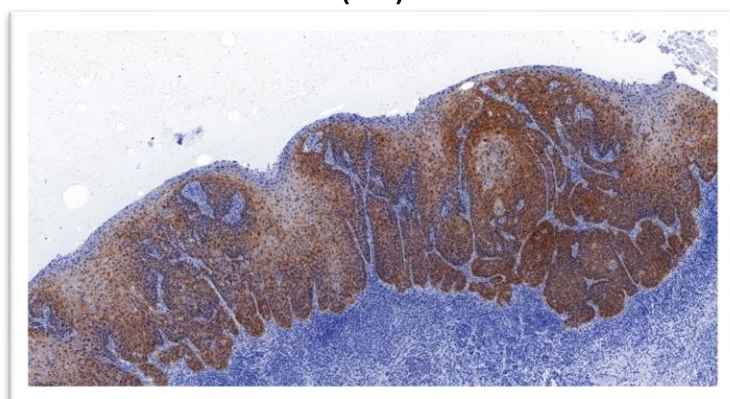

(49)

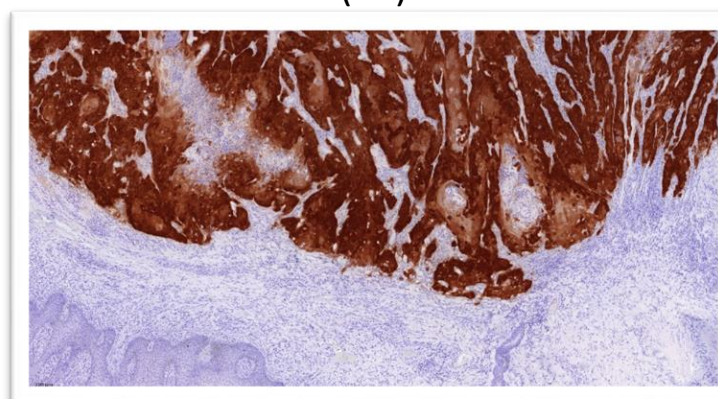

(52)

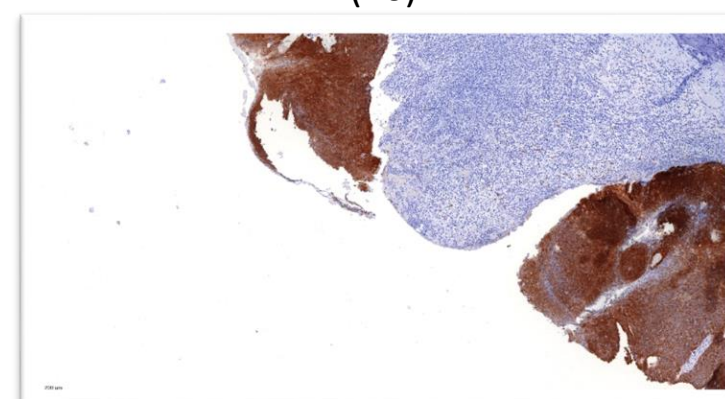

(54)

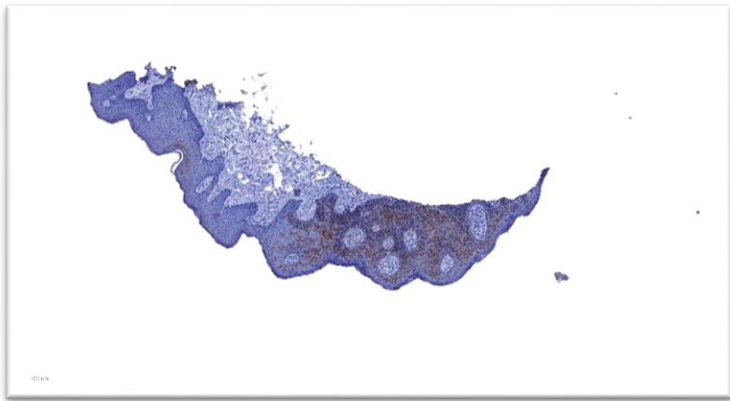

(55)

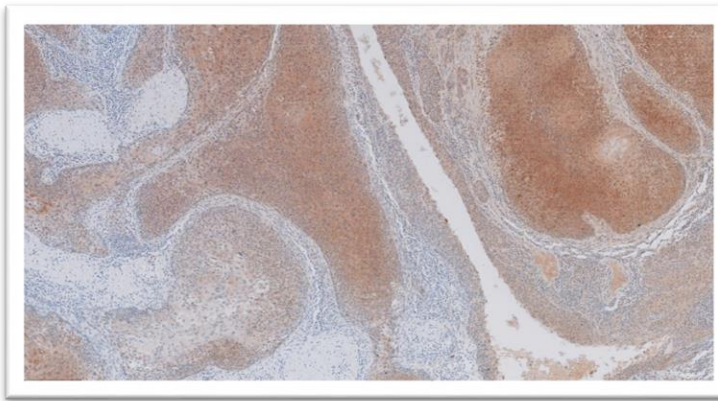

(56)

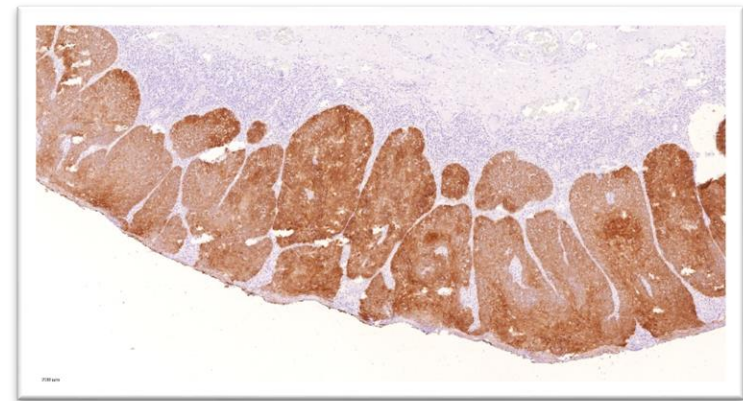

(57)

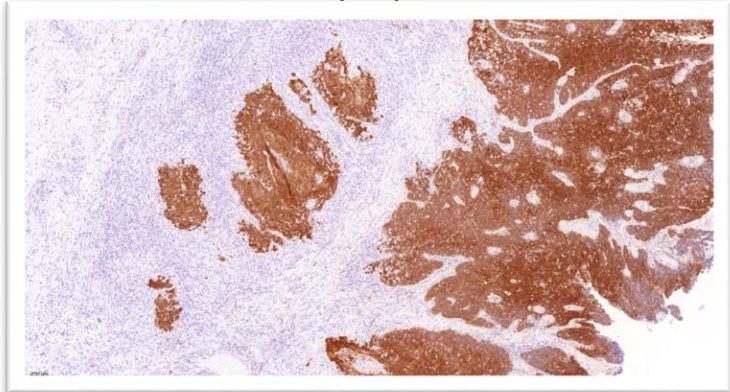

(60)

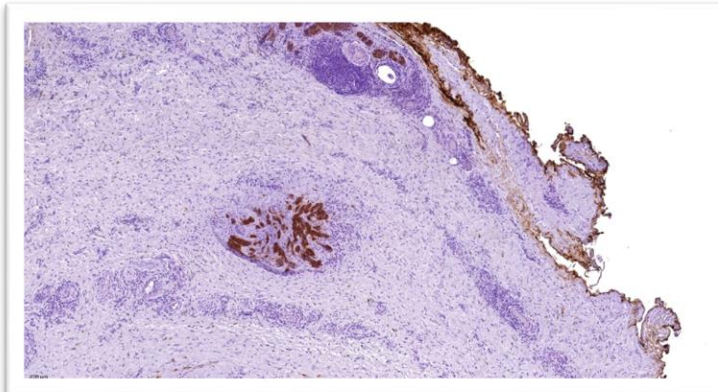

(63)

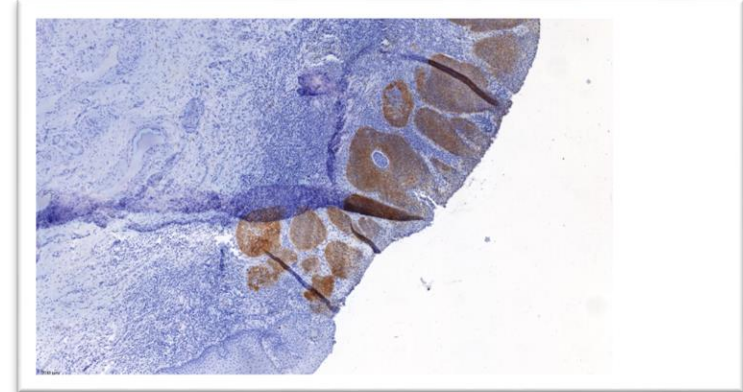

(65)

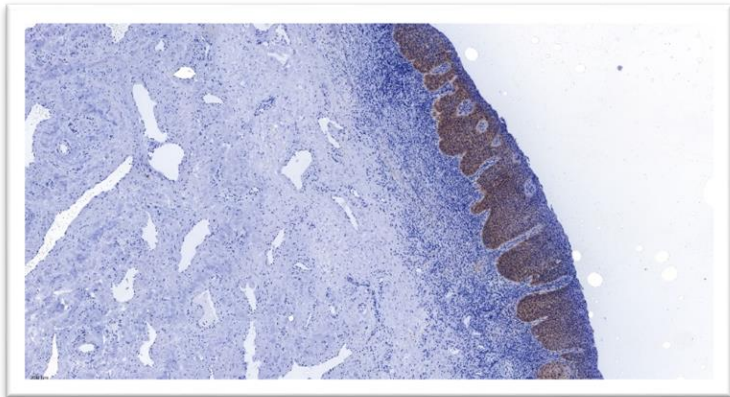

(66)

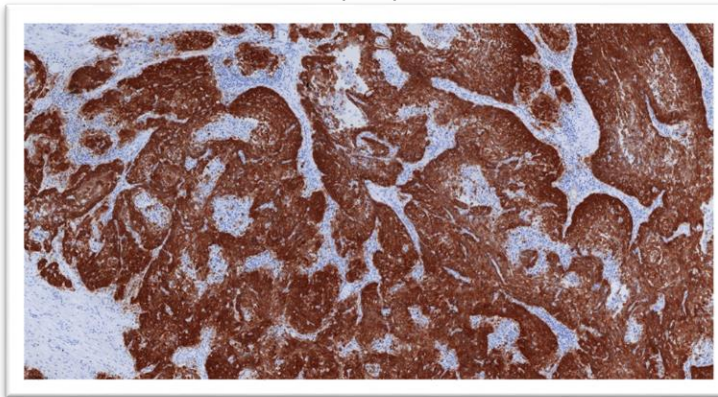

(68)

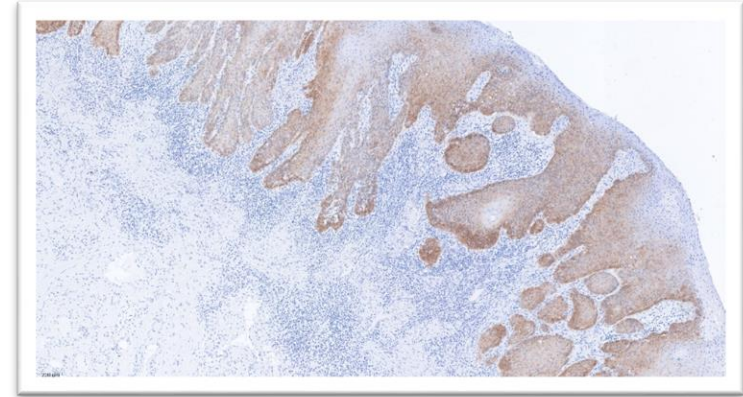

(70)

Supplement: Supplementary file 2 — Additional file 2: Fig. 2. Positive p16 stainings. [file 12894_2022_962_MOESM2_ESM.pdf]

## Slide 1
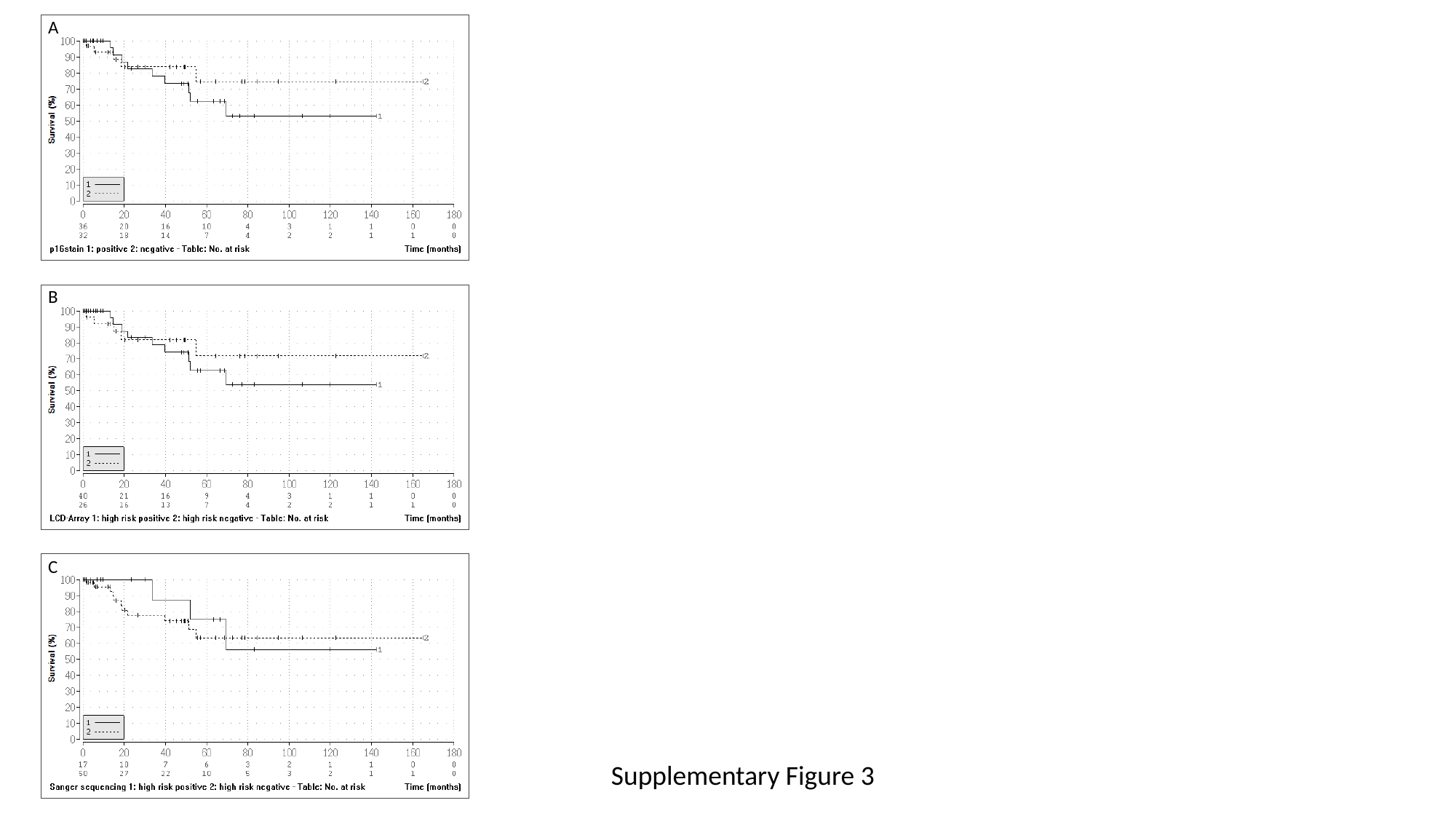

A
B
C
Supplementary Figure 3

Supplement: Supplementary file 3 — Additional file 3: Fig. 3. A Kaplan–Meier-Estimator for p16 positive and negative staining for overall survival in the cohort of penile neoplasias (p = 0.43; Log-rank-test, Cox-Mantel and Peto-Pike). B Kaplan–Meier-Estimator for LCD-Array with regard to HPV high risk positive and HPV high risk negative cases for overall survival in the cohort of penile neoplasias (p = 0.59; Log-rank-test, Cox-Mantel and Peto-Pike). C Kaplan–Meier-Estimator for Sanger sequencing with regard to HPV high risk positive and HPV high risk negative cases for overall survival in the cohort of penile neoplasias (p = 0.62; Log-rank-test, Cox-Mantel and Peto-Pike). [file 12894_2022_962_MOESM3_ESM.pptx]
