# Supplementary material for: The routine use of LCD-Array hybridisation technique for HPV subtyping in the diagnosis of penile carcinoma compared to other methods
Source: BMC Urol. 2022 Jan 29;22:10. doi: 10.1186/s12894-022-00962-4 (PMC8801096; doi:10.1186/s12894-022-00962-4)
Supplement: Supplementary file 4 — Additional file 4: Table 1a. HPV status of invasive squamous cell carcinomas by p16 immunohistochemistry with regard to tumor characteristics in evaluable cases**. Table 1b. HPV status by Sanger sequencing technique with regard to tumor characteristics in evaluable cases**. [file 12894_2022_962_MOESM4_ESM.docx]

Supplementary Table 1a

| HPV status of invasive squamous cell carcinomas by p16 immunohistochemistry with regard to tumor characteristics in evaluable cases** | | | |
| --- | --- | --- | --- |
|  |  |  |  |
| Characteristics | p16 negative (n) | p16 positive (n) | p*** |
| pTX | 2 | 0 | 0,14 |
| pT1a | 10 | 13 |  |
| pT1b | 3 | 4 |  |
| pT2 | 14 | 9 |  |
| pT3 | 0 | 3 |  |
| pT4 | 0 | 2 |  |
| pNX | 14 | 24 | 0,04 |
| pN0 | 10 | 3 |  |
| pN1, pN2, pN3 | 5 | 4 |  |
| LX | 1 | 0 | 0,75 |
| L0 | 23 | 24 |  |
| L1 | 5 | 7 |  |
| VX | 1 | 0 | 0,49 |
| V0 | 25 | 29 |  |
| V1 | 3 | 2 |  |
| PnX | 1 | 0 | 0,576 |
| Pn0 | 25 | 28 |  |
| Pn1 | 3 | 3 |  |
| G1 | 10 | 2 | 0,023 |
| G2 | 16 | 23 |  |
| G3 | 3 | 6 |  |
| usual morphology | 27 | 27 | 0,672 |
| basaloid morphology | 2 | 4 |  |
| infiltrative pattern | 17 | 21 | 0,593 |
| pushing borders | 12 | 10 |  |
|  |  |  |  |
| ** only cases are reported with known tumour characteristics | | | |
| ***Fisher-Freeman-Halton's exact contingency table-test | | | |

Supplementary Table 1b

| HPV status by Sanger sequencing technique with regard to tumor characteristics in evaluable cases** | | | |
| --- | --- | --- | --- |
|  |  |  |  |
| Characteristics | HPV high risk negative (n) | HPV high risk positive (n) | p*** |
| pTX | 2 | 0 | 0,120 |
| pT1a | 14 | 9 |  |
| pT1b | 4 | 3 |  |
| pT2 | 20 | 2 |  |
| pT3 | 2 | 1 |  |
| pT4 | 2 | 0 |  |
| pNX | 23 | 14 | 0,010 |
| pN0 | 13 | 0 |  |
| pN1, pN2, pN3 | 8 | 1 |  |
| LX | 1 | 0 | 0,785 |
| L0 | 33 | 13 |  |
| L1 | 10 | 2 |  |
| VX | 1 | 0 | 1,000 |
| V0 | 39 | 14 |  |
| V1 | 4 | 1 |  |
| PnX | 1 | 0 | 0,488 |
| Pn0 | 38 | 15 |  |
| Pn1 | 5 | 0 |  |
| G1 | 10 | 2 | 0,902 |
| G2 | 28 | 11 |  |
| G3 | 6 | 2 |  |
| usual morphology | 40 | 13 | 0,638 |
| basaloid morphology | 4 | 2 |  |
| infiltrative pattern | 29 | 8 | 0,537 |
| pushing borders | 15 | 7 |  |
|  |  |  |  |
| ** only cases are reported with known tumour characteristics | | | |
| ***Fisher-Freeman-Halton's exact contingency table-test | | | |
